# Upcycling recycled PET into a conductive electrode for a molecularly imprinted polymer based electrochemical sensor for 17β-estradiol detection

**DOI:** 10.1039/d6ra09742a

**Published:** 2026-11-10

**Authors:** Muhammad Faran Tahir, Tiwa Yimyai, Jan D'Haen, Ronald Thoelen, Anitha Ethirajan

**Affiliations:** a UHasselt, Institute for Materials Research (IUMAT) Martelarenlaan 42 Hasselt B-3500 Belgium; b imec, IUMAT Wetenschapspark 1 Diepenbeek B-3590 Belgium anitha.ethirajan@uhasselt.be

## Abstract

The upcycling of plastic waste into advanced materials has emerged as a promising strategy across various technologies, including the development of next-generation sensors. This sustainable approach promotes green technological development. In this work, we transformed recycled polyethylene terephthalate (rPET) into a functional conductive electrode substrate for a molecularly imprinted polymer (MIP) based electrochemical sensor for the detection of 17β-estradiol (E2). The latter also serves as a relevant model compound as a pollutant to study the sensor's applicability in complex matrices. The MIP-based sensor was fabricated by the electropolymerization of an *o*-phenylenediamine (*o*-PD) monomer in the presence of E2 template molecules and gold(iii) chloride trihydrate onto gold-coated rPET substrates. The physical properties of MIP films were characterized by scanning electron microscopy (SEM), atomic force microscopy, contact angle measurement, cyclic voltammetry, and electrochemical impedance spectroscopy. The binding of E2 molecules to the MIP surface hindered electron transfer of the redox probes, resulting in a concentration-dependent suppression of the electrochemical current measured by square wave voltammetry. The sensor showed high affinity for E2 molecules with a dissociation constant (*K*_d_) of 11.2 nM over a linear detection range of E2 concentrations (0.5–500 nM), and a limit of detection (LOD) of 0.18 and 0.41 nM in PBS and in wastewater samples without any complex pretreatment steps, respectively. This developed MIP-based sensor demonstrated the potential of alternative sensors for real-world environmental monitoring of trace endocrine disruptors, while successfully converting rPET into valuable technological products offers a practical roadmap to accelerate the development of sustainable and circular electronics.

## Abstract 2

A recycled PET based conductive electrode incorporating a gold-doped molecularly imprinted polymer for selective electrochemical detection of 17β-estradiol in wastewater. Created in BioRender.

## Graphical abstract

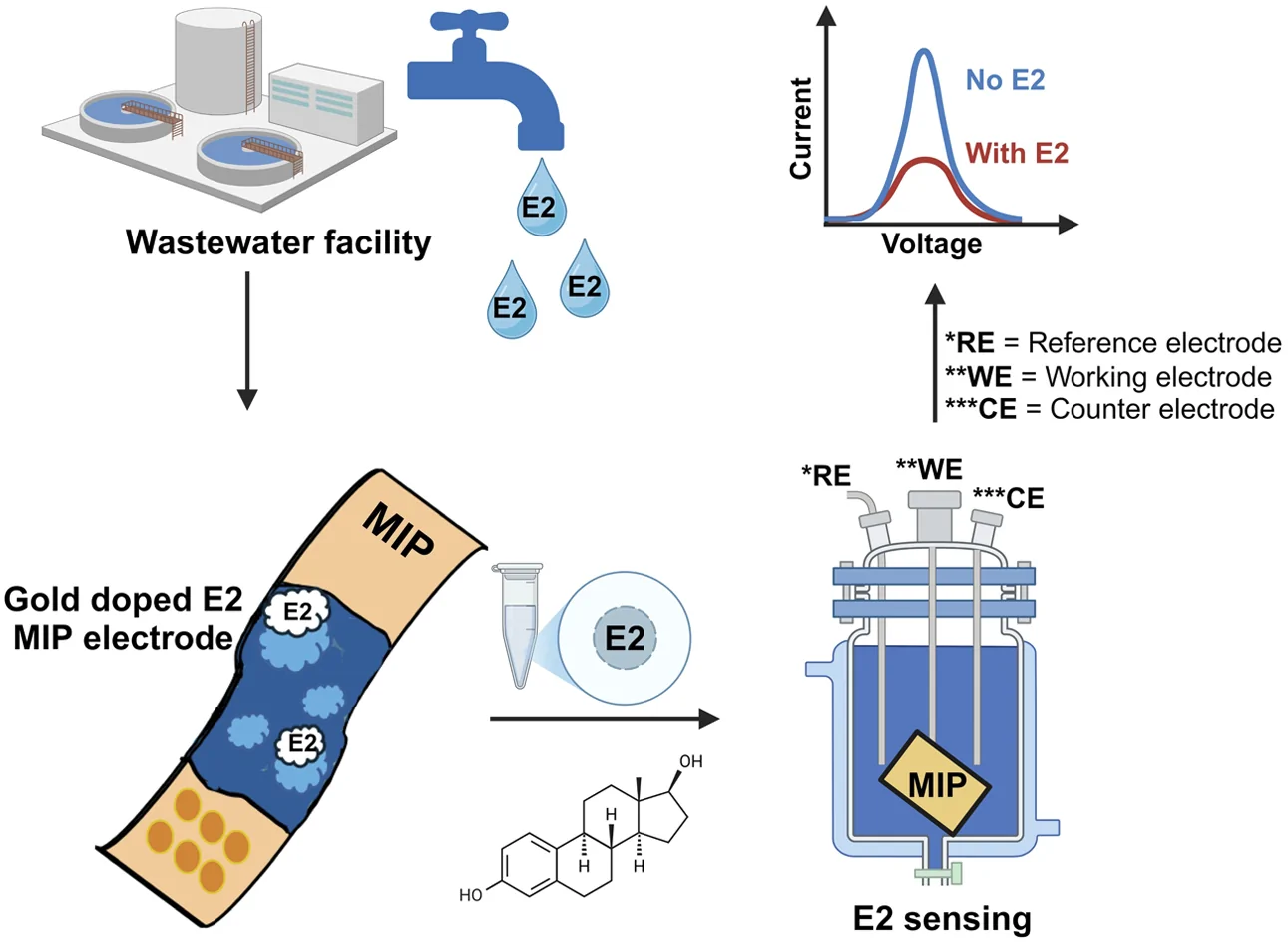

## 1. Introduction

In the electronics and sensor industries, polymer films have been widely utilized as reliable substrates or device components owing to their light weight, low cost, flexibility, mechanical robustness, and ease of mass production.^1–3^ For instance, polyimide (PI) films, commonly known as commercial Kapton®, are commonly employed in flexible printed circuit boards (PCBs) owing to their thermal stability. Furthermore, many research studies have employed various polymer films for sensor development, such as polyethylene terephthalate (PET),^4–8^ polyimide (PI),^9^ polyethylene naphthalate (PEN),^10^ polydimethylsiloxane (PDMS).^11^ Among these polymers, polyethylene terephthalate (PET) stands out as one of the most extensively utilized materials beyond electronic devices, as it is employed in products ranging from beverage packaging, textile fibers, and toys to everyday consumer goods. Consequently, the exponential accumulation of PET waste has escalated into a critical global environmental crisis, causing severe threats to ecosystems worldwide. To mitigate this ecological impact, recycling strategies have been implemented to reprocess post-consumer PET. However, the practical recycling process relies on mechanical re-melting, which can lead to downcycling owing to the thermal degradation of the polymer matrix,^12,13^ which yields low-tier products with low market value. To overcome this limitation, recent studies have focused on utilizing recycled PET (rPET) for upcycling, aiming to turn this post-consumer PET into functional conductive electrode substrates for products such as electronic devices, batteries, and sensors.^14,15^ In recent years, the development of next-generation electronic devices and sensors has increasingly gained attention for the upcycling strategy.^16^ Rather than only focusing on achieving high performance, this strategy balances environmental sustainability with economic viability by simultaneously reducing environmental burdens and generating commercial benefits.

Apart from the challenges of PET waste management, environment and human health have faced another big threat from chemical pollution, especially water resources contaminated with endocrine disrupting chemicals (EDCs) such as steroid hormones.^17–19^ 17β-Estradiol (E2) is a steroidal endocrine disruptor which can lead to chronic diseases such as endocrine dysfunction, male infertility, and breast and testicular cancers.^20^ The continuous, uncontrolled release of E2 in water sources has reduced water quality by interrupting the balance of aquatic ecosystems.^21^ Thanks to its limited degradation in the environment, E2 exhibits prolonged persistence and potential accumulation, which contribute to increased pollution levels and toxic effects in ecosystems.^22^ The concentration of E2 in aquatic environments is highly variable and depends on several factors, such as wastewater origin, the efficiency of treatment, and the proximity to, *e.g.*, dense population and livestock locations.^23^ For example, E2 concentrations of 175 ng L^−1^ (∼0.64 nM) have been reported in surface water in Italy, whereas concentrations of 125 ng L^−1^ (∼0.46 nM) have been observed in wastewater treatment streams in Canada.^24^ Moreover, higher levels reaching 0.73 nM have been observed in municipal sewage water in the Czech Republic,^25^ while concentrations as high as 9.73 nM have been detected in wastewater treatment plant effluents in Mexico.^26^ Taken together, such variabilities highlight the critical requirement for monitoring to control environmental discharge within regulatory thresholds.

Traditionally, the detection of this trace chemical relies on standard laboratory instrumentation such as immunoassays,^27^ liquid chromatography-mass spectrometry (LC-MS), high-performance liquid chromatography (HPLC), and gas chromatography-mass spectrometry (GC-MS).^28^ Although they are effective and sensitive readout methods for detecting trace amounts of analytes, these techniques are time-consuming and require complex sample pretreatment and trained personnel to execute.^29^ Therefore, alternative sensor technologies that are rapid, cost-effective, and capable of selective sensing are needed to broaden practical applications in environmental monitoring and wastewater surveillance.

Molecularly imprinted polymers (MIPs) have emerged as promising alternative sensing platforms for the detection of small molecules and environmental pollutants owing to their versatile customization in detecting diverse analytes, stability, low cost, possible portability for on-site monitoring, and minimal sample requirements.^19,30^ MIPs are capable of binding target molecules with strong binding affinity and selectivity *via* the specific shape and size of imprinted cavities and template–polymer interactions.^31,32^ The MIPs can be synthesized by incorporating template molecules (typically target analytes) into functional monomers (single or multiple types) *via* different polymerization techniques such as electro-, thermal-, and photopolymerizations.^33^ The template molecules embedded in the polymer matrix were employed to facilitate the formation of complementary empty cavities, which are later formed as binding sites after the templates were removed. The arrangement of template molecules and functional monomers during polymerization influenced the characteristics (size, shape, and molecular interactions) of resulting binding sites, ensuring rebinding capabilities (specificity and selectivity) for the target analyte, and generating measurable signals *via* appropriate readout techniques.^34,35^

Previous research has successfully developed MIP-based sensors for detecting steroid hormones^36–39^ using electrochemical techniques such as differential pulse voltammetry (DPV), cyclic voltammetry (CV), electrochemical impedance spectroscopy (EIS), linear sweep voltammetry (LSV), and square wave voltammetry (SWV). The electrochemical techniques offer high sensitivity and real-time readouts, making these MIP-based sensors more suitable and practical for detecting low concentrations of analytes in complex media. However, the MIP-based sensors were typically fabricated on rigid electrode substrates such as gold,^40^ glassy carbon,^41^ and silicon.^38,39,42^ While these conventional substrates provide excellent electrochemical sensitivity, they are likely constrained by high manufacturing cost, difficulty to handle during the fabrication process because of brittleness, besides they are difficult to recycle or reprocess after their operational lifetime. Consequently, the introduction of polymer films as electrode substrates for MIP-based sensors offers competitive advantages such as lightweight, flexibility, and compatibility with scalable manufacturing.

By intertwining the upcycling strategy and next generation MIP-based sensor development, this work demonstrated the conversion of a commercially available rPET film into a functional conductive electrode substrate for the development of a MIP-based sensor. Since the rPET is non-conductive, a thin layer (∼200 nm) of gold was deposited onto the rPET film *via* thermal evaporation to obtain a conductive working electrode. The gold-coated rPET electrode is particularly well suited for electrochemical MIP-based sensors owing to their excellent electrical conductivity, chemical stability in aqueous environments, and compatibility with surface functionalization and electropolymerization. Moreover, the rPET electrode offers the distinct advantage of being lightweight and conformity which lowers transportation costs and reduces structural stress on integrated systems. To the best of our knowledge, no reports have been published for the MIP-based electrochemical sensor utilizing rPET as an electrode substrate.

Herein, we developed an upcycled gold-doped MIP-based electrochemical sensor through electropolymerization for rapid and selective detection of E2 in wastewater. The flexible planar rPET film coated with a gold layer was used as a working electrode (WE). *o*-Phenylenediamine (*o*-PD) functional monomer was polymerized in the presence of gold(iii) chloride trihydrate (HAuCl_4_·3H_2_O) and E2 template molecules onto the gold-coated rPET substrates to obtain a MIP sensor. The MIP layer of poly(*o*-PD), consisting of abundant amine groups, could enhance binding affinity through physical interaction with hydroxyl groups of E2 target molecules *via* hydrogen bonding. The gold nanoparticles (AuNPs) in the MIP layer are expected to enhance the surface area and provide an electroconductive pathway, thereby facilitating efficient charge transfer of the redox probes occurring at the MIP layer through the gold surfaces,^43^ for an improved electrochemical response and increased sensitivity of the sensor. Electrochemical sensing performance was evaluated by exposing the MIP sensor to phosphate buffer solutions (PBS) and wastewater solutions spiked with various concentrations of E2. The developed MIP sensor exhibited reliable selectivity and sensitivity toward E2, confirming successful imprinting and effective analyte recognition. Furthermore, utilizing rPET as an electrode demonstrates the upcycling of discarded PET waste, providing a sustainable supply chain for developing sensors for wastewater monitoring.

## 2. Experimental section

### 2.1 Chemicals and materials

17β-Estradiol (E2) (≥98%), *o*-phenylenediamine (*o*-PD), potassium ferricyanide (K_3_[Fe(CN)_6_], 99%), and sodium acetate were purchased from Sigma-Aldrich. Estrone (E1, ≥99%), estriol (E3, 97%), potassium ferrocyanide (K_4_[Fe(CN)_6_], 99%), and phosphate-buffered saline (pH 7.4) packs were purchased from Thermo Scientific. Gold(iii) chloride trihydrate (HAuCl_4_·3H_2_O), acetic acid, and acetonitrile were acquired from VWR Chemicals, and potassium chloride (KCl) was purchased from Merck. rPET films (thickness = 75 microns) were purchased from TEKRA, LLC, with post-industrial content of up to 50% of the total composition. These rPET films exhibit high mechanical strength (tensile strength up to ∼186 MPa), good flexibility (elongation up to ∼110%), and thermal stability with limited shrinkage at elevated temperatures. Wastewater influent samples were obtained from Aquafin Hasselt and filtered with 2 µm Whatman filter paper to remove large debris and no further treatment was performed prior to sensor measurements. Ferro-/ferricyanide redox couple solution was prepared from a mixture of 5 mM K_3_[Fe(CN)_6_]/K_4_[Fe(CN)_6_] (1 : 1 M ratio) in 0.1 M KCl. All chemicals were of analytical grade and used without further purification. A laser cutter (FLUX Beamo, Belgium) was used to obtain a uniform size of rPET substrates.

### 2.2 Preparation of the molecularly imprinted polymer (MIP) sensor

The MIPs were deposited on the gold-coated rPET films *via* electropolymerization. First, the key parameters for MIP fabrication were systematically optimized to ensure the maximum electrochemical sensitivity of MIP sensors. The corresponding parameters include electropolymerization cycles, the effect of the pH of the acetate buffer solution, monomer-to-template ratio, washing time, eluents to remove the E2 molecule, and incubation time. Each parameter was investigated sequentially by varying its value, while the other parameters were maintained at a constant value (see details in the SI). The optimal parameters were used for the preparation of MIP sensors, which were subsequently employed for the sensing investigations. The optimized parameters and protocol are described here.

A 5 cm × 5 cm rPET film (shown in Fig. S1a) was cleaned by immersing it in ethanol under ultrasonication for 15 minutes and then rinsed with excess deionized (DI) water. A gold layer with a thickness of 200 nm was deposited on the rPET films *via* the thermal evaporation technique to obtain a flexible gold-coated rPET substrate. Consequently, the gold-coated substrates were laser-cut into 1 cm × 1 cm pieces. MIP layers were electrodeposited on these gold-coated rPET substrates (shown in Fig. S1b) by cyclic voltammetry in a solution containing 5 mM *o*-PD in 0.1 M acetate buffer solution, pH 4 (10 mL), 0.5 mM E2 template molecule in ethanol (5 mL), and 0.1 mM HAuCl_4_ in 0.1 M KCl solution (5 mL). In order to assess the specific binding of the target molecules, non-imprinted polymer (NIP) layers were electrodeposited following the same procedure as the MIP layers but without E2 template molecules. The optimal formulation used for the synthesis of MIP/NIP electrodes is mentioned in Table S1. The electropolymerization was carried out by applying a cyclic potential from 0 to 1 V *vs.* Ag/AgCl at a scan rate of 50 mV s^−1^ for 25 cycles. To remove E2 template molecules, the electropolymerized MIP on gold-coated substrates (MIP electrode) was soaked in acetonitrile for 10 min (see the details for the optimization of eluents for template removal and washing time in the results and discussion section (confer *vide infra*) and in the supplementary information) under gentle shaking at 100 rpm on a rotational shaker, followed by drying at room temperature to obtain MIP sensors. NIP electrodes treated identically were used as control samples.

Herein, the gold-coated rPET substrate is referred to as the bare electrode; the MIP on the gold-coated substrate, electropolymerized in the presence of gold chloride trihydrate, is referred to as the MIP electrode. Whereas the MIP electrode electropolymerized in the absence of gold chloride trihydrate is denoted as MIP_0_ electrode. Following the template removal process, the MIP electrode is designated as the MIP-E2 sensor, whereas the MIP_0_ electrode is denoted as the MIP_0_-E2 sensor. Similarly, electrodes prepared without E2 template molecules after electropolymerization are referred to as NIP electrode, and after template removal, it is called NIP sensor.

### 2.3 Sensor characterization

Cyclic voltammetry (CV), square wave voltammetry (SWV), and electrochemical impedance spectroscopy (EIS) were performed to characterize MIP/NIP sensors in the presence of redox probes by utilizing a potentiostat (PalmSens4, Palmsens BV, the Netherlands) with a three-electrode system. Gold-coated rPET substrates or MIP/NIP sensors were used as working electrodes (WE); platinum wire (thickness 0.5 mm) was used as a counter electrode, and Ag/AgCl (saturated in KCl) was used as a reference electrode. A PDMS *O*-ring was employed in the flow cell to prevent leakage and to precisely define the sensing area as 28 mm^2^. The distance between the working and counter electrode was 1.7 mm.

The sensing performance was evaluated by incubating MIP-E2 and NIP sensors in PBS and wastewater solutions, which were spiked with various concentrations of E2 for 10 minutes. To obtain the data for plotting the calibration curve, each sensor was incubated in a specific E2 concentration. The electrochemical measurements were performed by CV with a potential range of 0–0.5 V *vs.* Ag/AgCl at a scan rate of 100 mV s^−1^, EIS by applying a zero-bias voltage and oscillating potential of 10 mV with the frequency ranging from 0.1 Hz to 100 kHz, and SWV with a potential range of 0–0.5 V *vs.* Ag/AgCl at a pulse size of 25 mV and frequency of 10 Hz. EIS results were fitted with an equivalent circuit model by PSTrace software.

The morphology of polymeric MIP and NIP layers was characterized by a field emission gun scanning electron microscope (FEG–SEM) (ZEISS GeminiSEM 450 ZEISS, Germany). An atomic force microscopy (AFM) system (JPK–Bruker NanoWizard 3 Bruker, Germany) was used to measure the morphology and thickness of films with alternating current (AC) mode in air, with silicon ACTA tips from Scientec with a cantilever length of 125 µm, spring constant of 40 N m^−1^, and a resonance frequency of ∼300 kHz. AFM images were recorded over 5 µm × 5 µm scan areas with a resolution of 512 × 512 points. Morphological characterizations (SEM and AFM) were performed on three samples to evaluate surface uniformity. Electrochemical measurements for CV and SWV were carried out with three independent samples.

The thickness of the MIP and NIP layers was recorded *via* AFM with respective 3 independent samples by measuring the step height from the gold-coated rPET electrode to the polymerized MIP or NIP layer. The thickness is reported as the average and standard deviation of three measurements.

Contact angle experiments (sessile drops) were performed with three different independent samples with an instrument from OCA20 DataPhysics (Filderstadt, Germany) and SCA 20 software (Version V3.61.4). Water droplets of 3 µL were deposited on the surface of the bare gold-coated rPET, MIP, and NIP electrodes. The contact angle is reported as the average and standard deviation of three measurements.

## 3. Results and discussion

### 3.1 Preparation of MIP sensor

*o*-Phenylenediamine (*o*-PD) is widely used as a functional monomer in MIP sensors because of its amine (–NH_2_) groups, which form hydrogen bonds with template molecules, improving affinity and selectivity.^43,44^ Additionally, *o*-PD can be easily electropolymerized onto a gold substrate, producing stable ultrathin MIP films.^40,44^ HAuCl_4_ was selected to facilitate fast polymerization catalyzed by gold ions and improve sensitivity.^43^ The fabrication steps involved in the preparation of a MIP sensor are illustrated in Fig. 1. The MIP sensor was prepared by electropolymerization of *o*-PD in the presence of E2 template molecules on an electrode substrate. A three-electrode setup (working electrode (WE), counter electrode (CE), and reference electrode (RE)) was immersed in a pre-polymer mixture solution containing monomers, template molecules, and HAuCl_4_ in a glass flask. By applying a controlled potential range (0–1.0 V *vs.* Ag/AgCl), the *o*-PD monomers were oxidized and polymerized onto the electrode, forming a thin polymeric film that incorporated the template molecules. During the electropolymerization process, strong oxidizing agents, AuCl_4_^−^, react with the protonated *o*-PD, leading to the reduction of AuCl_4_^−^ ions to form gold nanoparticles (AuNPs),^43^ which act as active catalysts to facilitate the oxidative polymerization of *o*-PD monomers.^45^

**Fig. 1:**
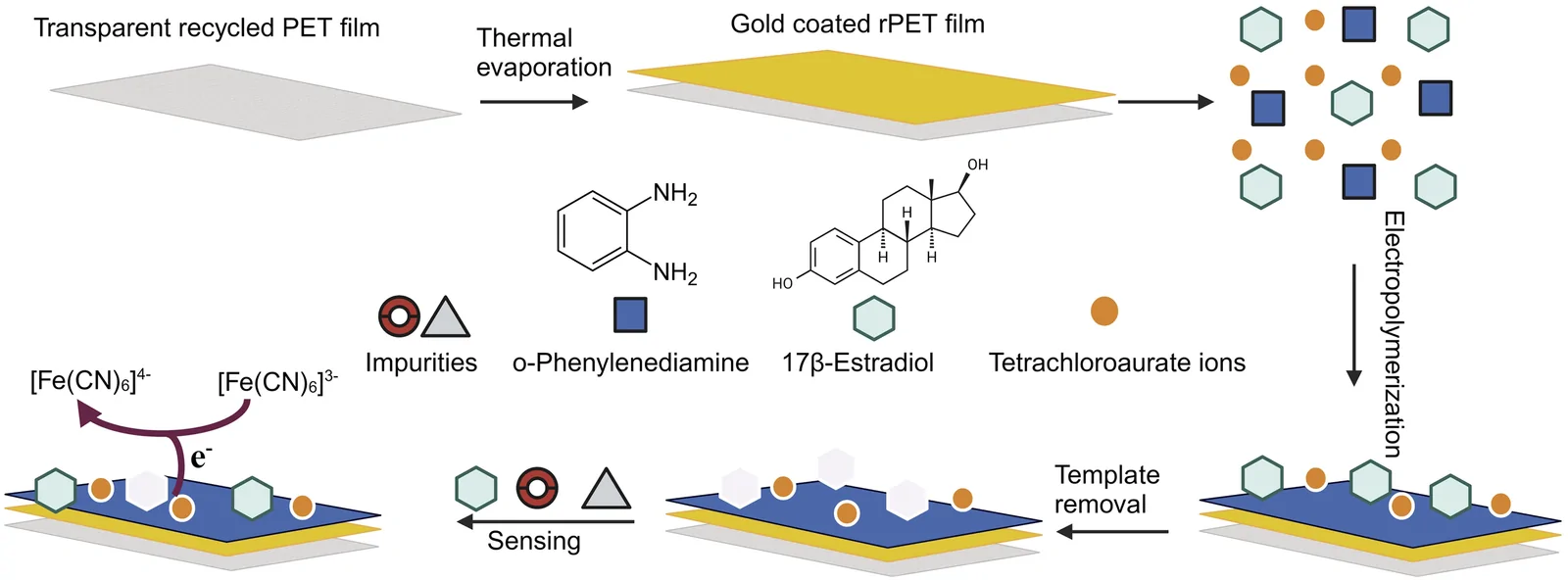
Schematic illustration showing the fabrication process and sensing mechanism of a MIP-E2 sensor. Created in BioRender.

The sensing performance of the MIP-based sensor, for instance, selectivity and sensitivity, is influenced by various factors during preparation, such as the CV electropolymerization cycle, pH of the polymerization electrolyte, monomer–template ratio, the choice of the eluent, the time required for the template extraction, and the incubation time of the target analyte to bind to the MIP sensor.^46^ Therefore, the corresponding parameters were systematically studied to ensure that the MIP sensor achieved the highest sensing ability (maximum current response). SWV was employed to quantify the oxidative peak current. The difference in oxidative peak current (Δ*I*) was determined to study the sensing ability of MIP sensors. Δ*I* corresponded to the difference between the current recorded after template removal (*I*_0_) and the current recorded after incubation in a PBS solution containing 100 nM of E2 (*I*_t_).

The number of CV electropolymerization cycles directly dictates the MIP film thickness, which is crucial for balancing sensor sensitivity and stability. Different MIP layers were prepared by varying CV cycles (15, 20, 25, 30, and 35). As shown in Fig. S2, a lower number of cycles (15 and 20 CV cycles) yielded an insufficiently thin layer, potentially lacking enough imprinted sites. Conversely, excessive cycles (30 and 35 cycles) led to an overly thick MIP film that hindered the diffusion of the redox probe to the electrode surface. Optimally, the maximum Δ*I* was achieved at 25 cycles. The effect of the polymerization electrolyte pH was subsequently investigated, as the pH significantly influences the functional monomer behavior and complex formation. The electropolymerization of *o*-PD is more favourable under acidic conditions, which facilitates the condensation of amino groups (–NH_2_) with the benzene ring of adjacent units to form a stable polymer network.^47,48^ The highest Δ*I* was achieved with a solution at pH 4 with 25 CV cycles (Fig. S3). However, at higher pH (pH 5), the level of conjugation decreases and gives less stability to the MIP films.^48^ Following the pH optimization, the molar ratio between monomer (*o*-PD) and template (E2) was investigated, as it governs the density and structural integrity of the recognition sites. The amount of template was fixed at 0.5 mM, and the amount of monomer was varied to 1, 5, 10, and 20 mM to investigate the highest Δ*I*. As shown in Fig. S4, the higher ratio of monomer-template ratio (10 : 0.5 and 20 : 0.5 mM) yielded thick MIP layers, which hindered efficient template removal and entrapped the template, resulting in fewer active binding sites. On the contrary, the lower monomer-template ratio of 1 : 0.5 mM provided an insufficient MIP layer to form well-defined imprinted sites.^49^ The highest Δ*I* was achieved with the monomer-template ratio of 5 : 0.5 mM. The washing time for template removal was investigated at different intervals (2, 5, 10, 15, 20, and 25 min). The maximum Δ*I* was achieved after 10 min of washing (Fig. S5), suggesting efficient removal of the E2 template. Conversely, a lower Δ*I* was observed after shorter washing time (2–5 min), indicating incomplete template extraction. This could be attributed to insufficient time to break the hydrogen bonding between the template and polymer.^50,51^ Subsequently, with the optimal washing time at 10 min, various eluents including phosphate-buffered saline (PBS), acetic acid: methanol (1 : 19), ethanol, and acetonitrile were evaluated to identify the most effective medium for E2 template removal. As shown in Fig. S6, the MIP sensor after washing with acetonitrile showed the highest Δ*I*, compared with other eluents. As reported in the literature, the electropolymerized poly(*o*-PD) is insoluble in acetonitrile.^52^ Therefore, the current change (maximum sensitivity) after washing with acetonitrile is attributed mostly to the elution of E2 template molecules. During the washing process, the eluents could induce swelling of the polymer matrix, thereby weakening the interaction (such as hydrogen bonding) between the template and polymer.^49^ This disruption in the polymer network caused the template molecule to detach, thus creating specific binding sites. As a next step, the MIP sensors prepared using the aforementioned optimal parameters (see details in the experimental section 2.2) were employed for the investigation of incubation time. The incubation study was performed by immersing the MIP-E2 sensor in PBS containing 100 nM E2 at different intervals (2, 4, 6, 8, 10, 12, and 14 min). As shown in Fig. S7, the Δ*I* increased markedly during the first 2–8 min of incubation due to the progressive rebinding of E2 molecules to the MIP sensors. After 10 min, the Δ*I* reached a plateau and remained nearly constant up to 14 min, indicating that equilibrium was achieved. Therefore, an incubation time of 10 min was selected for further investigations of sensing performance.

The electropolymerization of the MIP and NIP layers was monitored by cyclic voltammetry. The cyclic voltammograms displayed the oxidation peaks of the *o*-PD monomers in the first cycle of electropolymerization for MIP electrodes at 0.42 and 0.72 V *vs.* Ag/AgCl (Fig. 2a), consistent with a previous report for the electropolymerization of *o*-PD.^48^ Notably, the first oxidation peak of the MIP electrode (doped with AuNPs) occurred at a lower potential of 0.42 V *vs.* Ag/AgCl with a higher current of 388 µA, while a higher potential of 0.46 V *vs.* Ag/AgCl with a lower current of 243 µA was observed for the MIP_0_ electrode (Fig. S8a). These results confirmed that the presence of AuNPs influenced the electropolymerization process and enhanced film conductivity.^43^ In addition, with increasing scan cycles, a significant decrease in the oxidation peak current was observed, indicating the formation of insulating poly(*o*-PD) layers on the electrode surface (gold-coated rPET substrate), while random embedding of E2 template molecules within the polymer matrix occurred through hydrogen bonding between hydroxyl groups of E2 and protonated amine groups of poly *o*-PD-NH_3_^+^.^53^ The electropolymerization cyclic voltammograms of MIP and NIP electrodes were identical, indicating that the E2 template molecules did not affect the electropolymerization process of MIP samples. This is expected as E2 is not an electroactive molecule. These results confirmed the successful growth of polymer film on the gold surfaces.

**Fig. 2:**
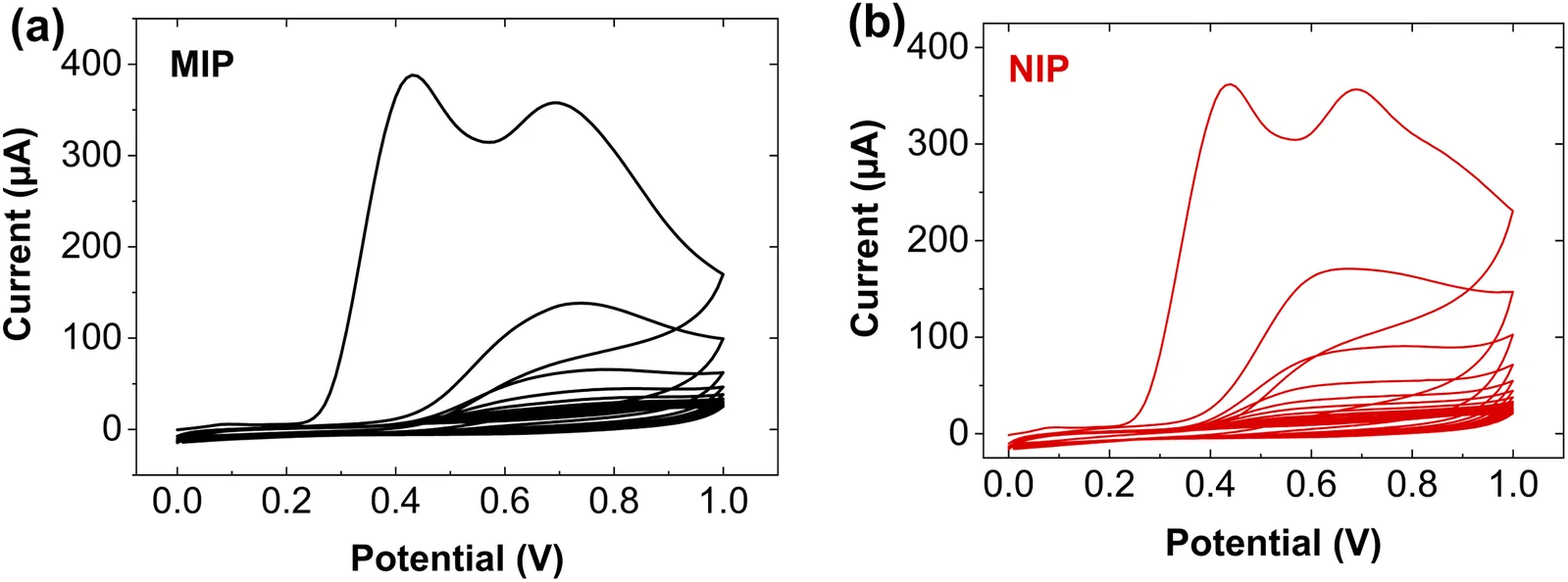
Representative cyclic voltammograms obtained during electropolymerization of (a) MIP and (b) NIP electrodes in a solution containing 5 mM *o*-PD in 0.1 M acetate buffer solution (pH 4), 0.5 mM E2 template molecule in ethanol (in case of MIP), and 0.1 mM HAuCl_4_ in 0.1 M KCl solution. The NIP electrode was prepared in the absence of E2 template molecules.

### 3.2 Surface and morphological characterization

The morphology, surface properties, and thickness of the samples were characterized by SEM and AFM. The surface wetting behaviour was studied by contact angle measurements. The SEM images showed that gold nanoparticles were dispersed randomly over the MIP/NIP electrode surfaces and showed distinct morphological differences (roughness) compared to the bare electrode (Fig. 3a–c). The roughness of the polymerized surfaces can be attributed to the rapid polymerization of *o*-PD monomers catalysed by gold nanoparticles.^43^ These results confirmed the successful deposition of the polymer layers with gold nanoparticles onto the bare gold surfaces.

**Fig. 3:**
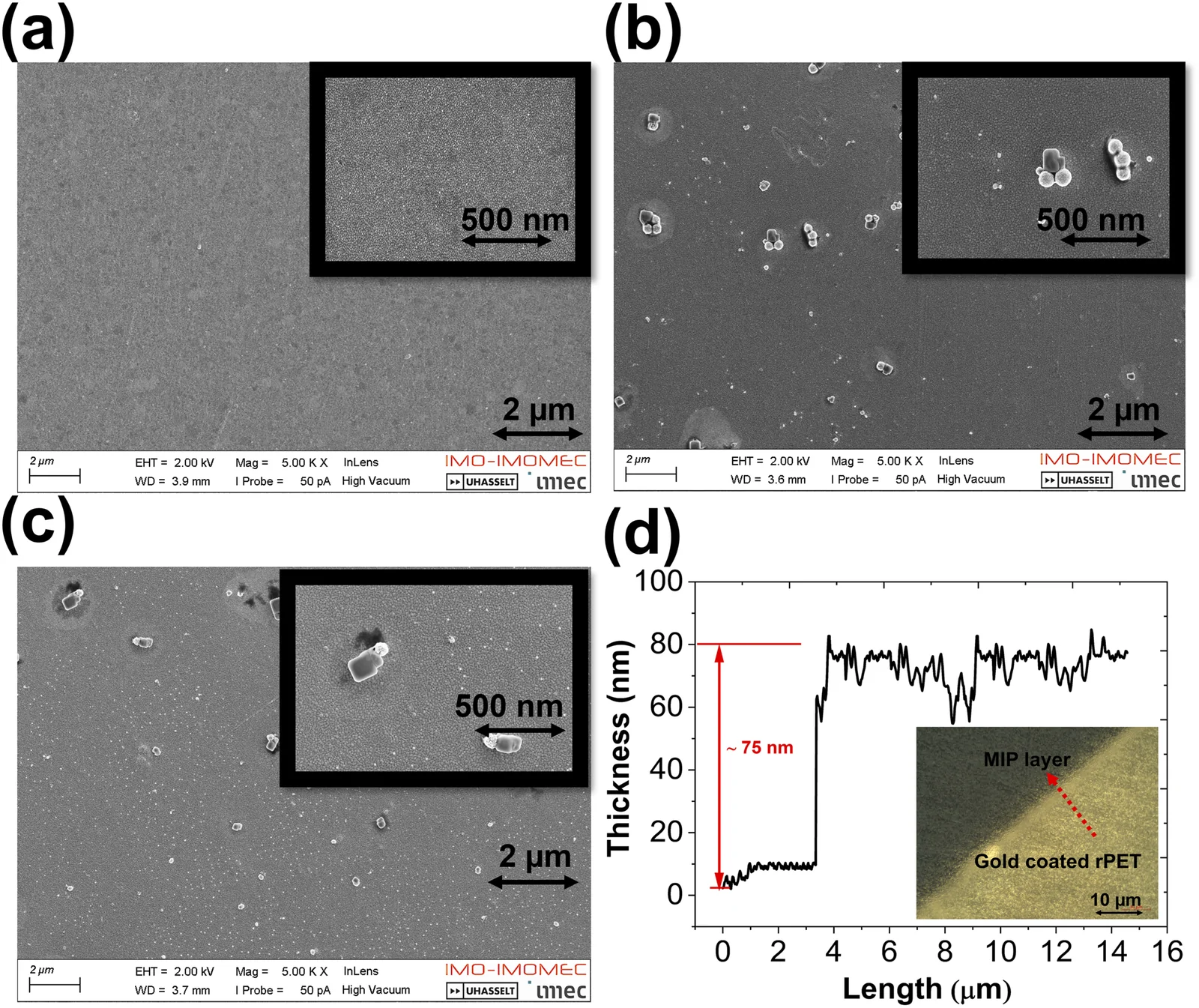
Scanning electron microscopy (SEM) images of (a) bare electrode, (b) pre-wash MIP electrode, and (c) pre-wash NIP electrode. The inset in a–c shows a high-magnification image of each electrode. (d) Representative step height profile (thickness) measured by AFM of the E2-MIP layer deposited on a gold-coated rPET substrate. The measurement was carried out along the dashed red line (inset).

The AFM results revealed that the MIP electrode had a rougher surface compared to the bare electrode (Fig. S9a–e). The root mean square (RMS) roughness of the bare electrode was 3.12 ± 0.01 nm, while that of MIP and NIP electrodes before washing was 17.71 ± 0.27 and 6.23 ± 0.03 nm, respectively. When comparing post-washing MIP and NIP electrodes, the MIP electrode showed greater surface roughness than the NIP electrode. This might be because of the presence of target molecules embedded within the polymer network.^54^ Surface roughness of the samples was smoother after washing of the template molecules, resulting in roughness of 12.90 ± 0.15 nm and 5.33 ± 0.08 nm for MIP and NIP sensors, respectively. The surface roughness of the NIP samples decreased by 14%, whereas the MIP samples exhibited a larger reduction of 27% after the extraction step. This greater decrease in the MIP samples is consistent with the removal of template molecules, although the washing process may also remove loosely bound structures, as supported by the SEM images before and after washing (Fig. S10), thereby contributing to the observed reduction in surface roughness.^54^ The morphological and surface properties of the fabricated sensors were in accordance with those reported in previous studies utilizing gold coated electrodes.^55–57^ The thickness of MIP and NIP layers before washing was 75 ± 1.3 nm (Fig. 3d) and 67 ± 0.89 nm, respectively, and decreased to 70 ± 0.9 nm and 65 ± 0.34 nm after washing. It is worth noting that the *o*-PD monomer is insoluble in acetonitrile^51^ (eluent used for E2 template removal); however, changes in surface roughness and thickness reduction might be influenced by the removal of template molecules.^58,59^ Contact angle results shown in Fig. S11 suggested a change in the surface properties of the samples. The electropolymerized MIP and NIP layers on the gold surface exhibited lower water contact angle (62.73° ± 1.02 and 57.01° ± 1.17, respectively) than that of bare gold (80.30° ± 0.5), which was attributed to the hydrophilicity of amino groups in poly(*o*-PD).^60^ A decrease in the water contact angle of the MIP sensor after washing (58.92° ± 0.12) can be attributed to the removal of the template molecule, whereas the NIP sensor showed a relatively small difference in contact angle after washing (55.31° ± 1.07).

### 3.3 Electrochemical characteristics

After the electropolymerization process, the prepared electrodes were subjected to electrochemical characterization to evaluate their performance and sensing behaviour. The characterization of MIP and NIP electrodes was performed by CV and EIS in a solution of 0.1 M KCl containing 5 mM redox probe of K_3_[Fe(CN)_6_]/K_4_[Fe(CN)_6_] with an equimolar ratio.

The cyclic voltammograms in Fig. 4a and b reveal that the oxidation peak current of the bare electrode is 167 µA at 0.3 V *vs.* Ag/AgCl (curve i), which subsequently decreased to 0.69 µA (curve ii) upon the formation of an insulating layer of MIP or NIP on the electrode surface. This insulating layer hindered charge transfer of redox probes reaching the gold electrode surface, resulting in a lower current response. The peak current of the MIP_0_ electrode was identical to that of the MIP electrode after polymerization (curve ii, Fig. S8b).

**Fig. 4:**
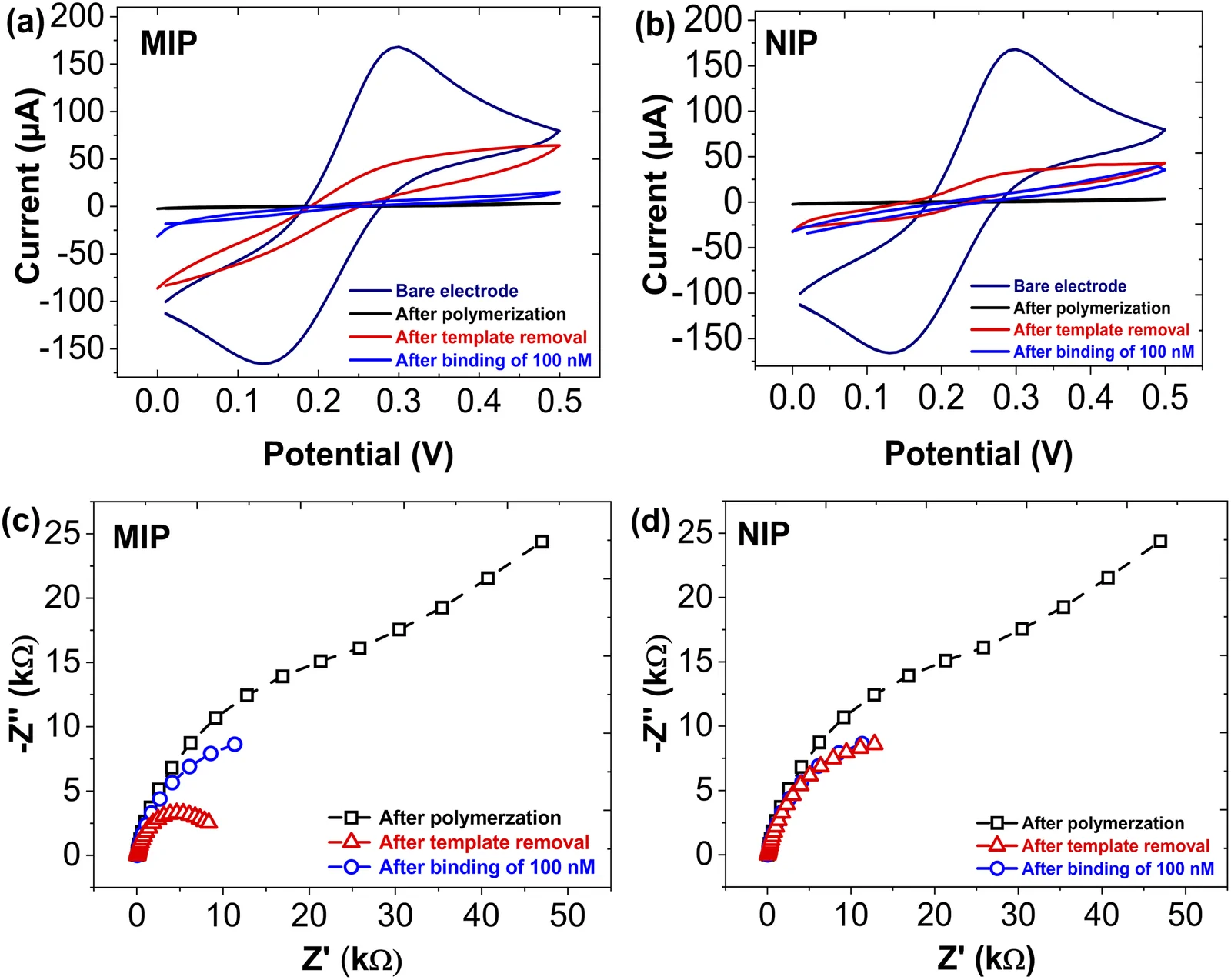
Cyclic voltammograms of (a) MIP and (b) NIP electrodes, including (i) bare electrode, (ii) after electropolymerization, (iii) after template removal, and (iv) after rebinding of E2 (100 nM in PBS). Nyquist plots obtained by electrochemical impedance spectroscopy (EIS) of (c) MIP and (d) NIP electrodes: (i) after polymerization, (ii) after template removal, and (iii) after rebinding of E2 (100 nM in PBS). All the experiments were performed in a 0.1 M KCl solution containing 5 mM redox probes.

Upon template removal using acetonitrile, the peak current of the MIP sensor increased to 59 µA at 0.3 V (curve (iii), Fig. 4a). Subsequently, subjecting the MIP-E2 sensor to 100 nM of E2 in PBS resulted in the peak current decrease to 13 µA (curve (iv), Fig. 4a), yielding a reduced oxidative peak current difference of 46 µA and suggesting that the rebinding of E2 molecules to the MIP sensor might hinder the charge transfer. In contrast, the MIP_0_-E2 sensors exhibited a lower peak current of 24 µA after template removal (curve iii, Fig. S8b), which decreased to 20 µA upon E2 binding (curve iv, Fig. S8b), demonstrating a smaller oxidative peak current drop of 4 µA than that of the MIP-E2 sensor exhibiting a significant current drop of 46 µA (from 59 to 13 µA upon E2 binding). These results demonstrated improved sensitivity (higher current difference) in the MIP-E2 sensor in the presence of AuNPs, which facilitates the charge transfer rate between the redox probes and the electrode.^43^ The peak current of the NIP electrode showed a similar trend in oxidative peak current as that of the MIP electrode for curves (i) and (ii) in Fig. 4b. After washing, the slight increase in oxidative current peak in case of NIP (curve (iii)) could be attributed to the removal of the loosely bound structures on the surface. This is also in agreement with the slightly reduced RMS roughness value observed in case of the NIP surfaces. However, the peak current of the NIP sensor did not change significantly after rebinding of 100 nM E2 (curve iv, Fig. 4b). Despite the theoretical absence of specific binding sites in the NIP layer, the decreased current is likely associated with the adsorption of E2 molecules on the gold surface, resulting in partial blockage of electroactive sites and consequently hindering the access of the redox probe to the electrode. The reduction in current response (resistance of charge transfer) could also be influenced by the Helmholtz double layer from E2 adsorption.^61^

Additionally, the electrochemical characteristics of MIP and NIP samples were investigated by EIS, which is a potential tool widely used to study the electrochemical properties of MIP-based biosensors.^38,39,43,45^ The impedance response is highly sensitive to interfacial changes occurring at the electrode–electrolyte boundary, making it suitable for studying changes at the electrode coated with MIP/NIP layers, such as the binding of the target molecules. The EIS results were consistent with the CV results. As shown in Fig. 4c, in the lower-frequency regime, the MIP-E2 sensor after template removal (curve ii) exhibited a lower impedance magnitude than the MIP electrode after electropolymerization (curve i). This suggests that the template acts as an insulating barrier and does not contribute to the overall charge transport within the MIP matrix. After incubation of the MIP electrode in an E2 solution (100 nM in PBS), the impedance amplitude increased (Fig. 4c, curve iii), indicating the rebinding of E2 molecules to the MIP sensor. This rebinding likely hindered electron penetration to the gold electrode surface and affected the capacitive behavior of the polymer–electrolyte interface. While the impedance amplitude of the MIP_0_-E2 sensor remained higher than that of the MIP-E2 sensor both after template removal and E2 rebinding (Fig. S8c), indicating an increased charge-transfer resistance due to the less conductive layer (MIP layer without AuNPs).

A decrease in the impedance amplitude for the NIP sensor after template removal (Fig. 4d curve (ii)) reflects the observation in the CV studies. Furthermore, the impedance of the NIP sensor remained unchanged after incubation in E2 solution (100 nM in PBS), indicating negligible binding of E2 on the NIP surface. The EIS results clearly demonstrated that the impedance of the MIP sensor increased after E2 rebinding, while the NIP sensor showed no change, confirming the effective binding of E2 on the MIP layer.

### 3.4 Analytical performance of MIP sensors

The analytical performance of the MIP-E2 sensor was evaluated by incubating the individual sensors in E2-spiked PBS with different concentrations (0.5, 2, 5, 10, 20, 50, 100, 300, and 500 nM). SWV was used as a readout to observe the oxidative peak current of MIP sensors after incubation in the E2-spiked PBS for 10 min. As shown in Fig. 5a, the SWV peak current at ∼0.23 V *vs.* Ag/AgCl of the MIP-E2 sensor decreased with the increase in E2 concentration, indicating rebinding of E2 molecules on the binding sites of the MIP-E2 sensor. While a negligible change in peak current was observed for the NIP sensor, implying insignificant adsorption of E2 molecules (Fig. 5b). The oxidative peak current difference (Δ*I*) was calculated and plotted against concentration (*C*) on a logarithmic scale (log_10_) of E2 to obtain calibration curves for MIP-E2 and NIP sensors (Fig. 5c). The calibration curves highlighted a clear distinction (even at the lowest concentration of 0.5 nM) of the current responses between MIP-E2 and NIP sensors, with linear equations of Δ*I* = 2.86 [*C*]+ 2.185 (*R*^2^ = 0.98, *n* = 3) and Δ*I* = 0.546 [*C*] + 0.91 (*R*^2^ = 0.95, *n* = 3), respectively. The MIP-E2 sensors showed higher sensitivity than that of NIP sensors over a broad detection range between 0.5 nM and 500 nM, with a limit of detection (LOD) of 0.18 nM. The LOD was calculated by the following equation:1
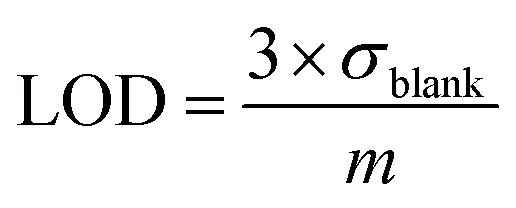
where *σ*_blank_ is defined as the standard deviation of the current response measured in blank (pure PBS) and calculated as 0.18 µA, and *m* is the slope of a linear equation, which is 2.86.

**Fig. 5:**
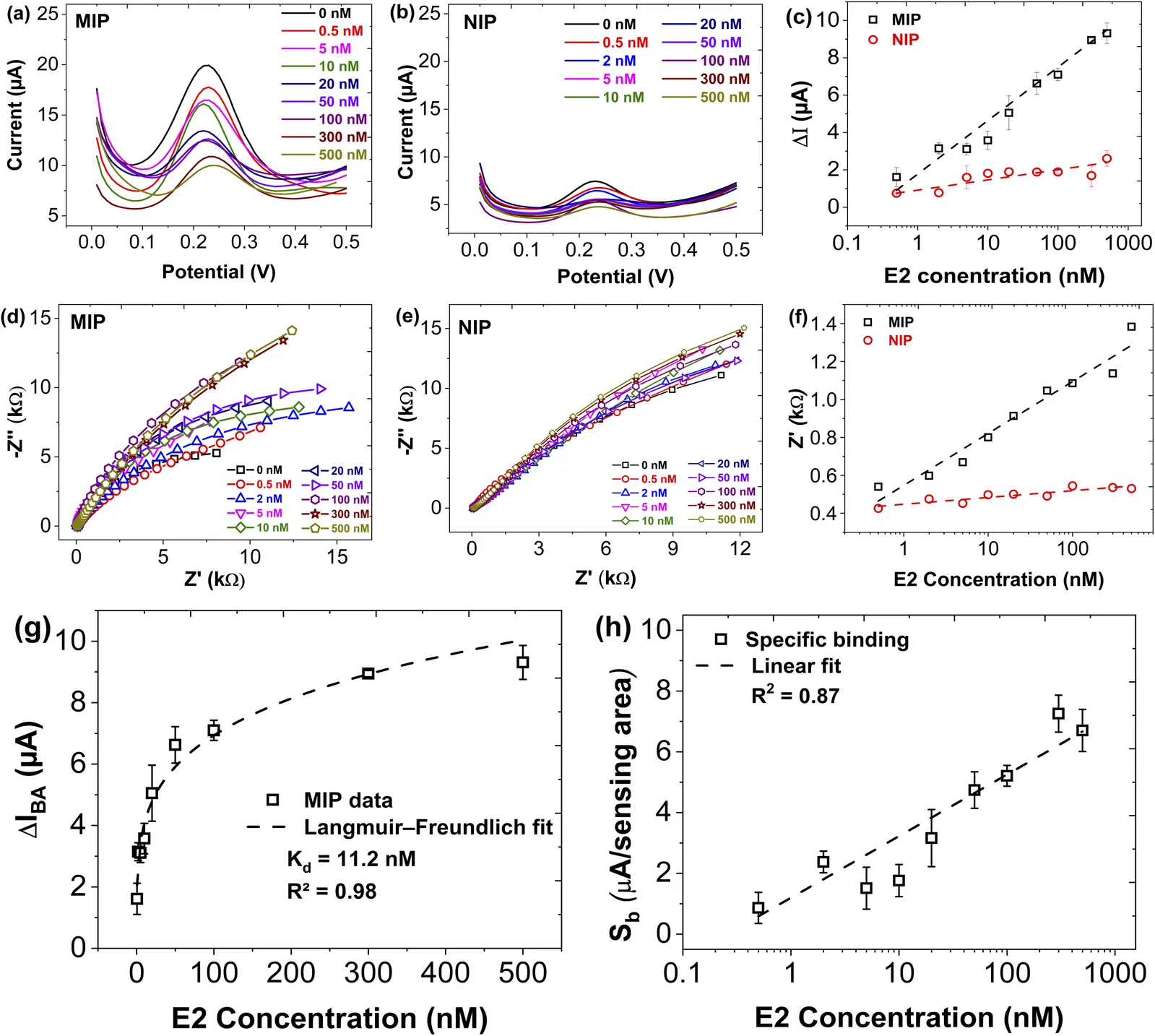
Square wave voltammograms for (a) MIP-E2 and (b) NIP sensors after incubation in different concentrations of E2 (0 to 500 nM in PBS). (c) Calibration curves for MIP-E2 and NIP sensors, *n* = 3. Nyquist plots for (d) MIP-E2 and (e) NIP sensors after incubation in different concentrations of E2 (0 to 500 nM in PBS). (f) Calibration curves plotted with real impedance (*Z*′) at 100 Hz against E2 concentration (0 to 500 nM in PBS) for MIP-E2 and NIP sensors. (g) The binding isotherm of E2, measured by Δ*I*_BA_ in SWV, was fitted by the Langmuir–Freundlich (LF) adsorption model, *n* = 3. (h) Specific binding curve plotted with SWV current difference between MIP-E2 and NIP sensors under a sensing area of 28 mm^2^, *n* = 3. All measurements were performed in a 0.1 M KCl solution containing 5 mM redox probes.

Notably, compared with the MIP_0_-E2 sensor, MIP-E2 sensors exhibited higher Δ*I* across the E2 concentration range between 0.5–500 nM (Fig. S8d), demonstrating that the incorporation of AuNPs promotes sensitivity.

The electrochemical sensing characteristic of the MIP-E2 sensor as a function of E2 concentration was also evaluated by the EIS technique. As shown in Fig. 5d, the impedance response at the lower frequency regime of MIP-E2 sensors increased with the increased E2 concentration, as evidenced by the enlargement of the semicircular region in Nyquist plots. The expanded semicircular diameter indicated a lower charge-transfer rate. The results were attributed to the binding of E2 molecules on the MIP sensors, which hindered redox probe molecules reaching through the gold electrode surface. In contrast, NIP sensors did not show a significant change in impedance (Fig. 5e), indicating that NIP sensors lacked specific binding sites for E2 molecules. The calibration curves in Fig. 5f were constructed with the *Z*′ at a frequency of 100 Hz. The operating frequency of 100 Hz was selected *a priori* based on the Bode phase response (Fig. S13a), where the phase angle reaches its maximum (∼65–70°), indicating a predominantly capacitive interfacial behaviour and optimal sensitivity to changes at the electrode–electrolyte interface. This frequency lies within the mid-to-low frequency region of the impedance spectrum, prior to the onset of diffusion-dominated (Warburg) contributions observed at lower frequencies, thereby ensuring a stable and sensitive readout.

The resulting curves revealed a clear and consistent trend, showing that *Z*′ of the MIP-E2 sensor increased proportionally with the E2 concentration. This linear relationship reflects the strong sensitivity of the sensor and its ability to effectively recognize E2 molecules through the imprinted sites. In contrast, the NIP sensor showed only minimal changes in response, confirming that the specific binding behavior observed in the MIP originates from the molecular imprinting process. Moreover, MIP-E2 sensors exhibited higher sensitivity than that of the NIP sensor, as evidenced by the linear calibration curves and their corresponding fitting equations of *Z*′ = 273.61[*C*] + 547.04 (*R*^2^ = 0.94) for MIP-E2 sensors, and *Z*′ = 35.67[*C*] + 447.61 (*R*^2^ = 0.80) for NIP sensors.

Physical and electrochemical characteristics of the MIP-E2 sensors could be explained through an equivalent circuit, which was fitted from Bode and Nyquist plots (Fig. S12 and S13). As shown in the equivalent circuit, *R*_s_ represents the ohmic electrolyte/series resistance, *R*_ct_ represents the effective interfacial charge-transfer resistance of the redox probe at the MIP-modified electrode, *C*_e_ represents the effective interfacial double layer capacitance of the MIP-modified electrode/electrolyte interface, and *W* is the Warburg element, which accounts for diffusion-related impedance, mainly associated with mass transport of the redox species/electrolyte through or near the MIP layer.^62^ The impedance response supports an interpretation in which E2 rebinding hinders redox-probe access and increases the effective interfacial impedance. At lower frequencies, the impedance was higher according to the dominance of *R*_ct_ caused by the binding of E2 (a non-conductive molecule) on the surfaces of the MIP-E2 sensor. The enlarged and non-ideal Nyquist response is consistent with increased interfacial resistance and distributed capacitive/diffusive behavior after E2 rebinding. The template binding likely reduces the permeability of the MIP layer and hinders electron transfer of the ferri/ferrocyanide redox probe, leading to increased *R*_ct_ and impedance. Moreover, the fitted data yielded a low chi-square value (*χ*^2^) of 9 × 10^−4^ and 3 × 10^−4^ for Bode and Nyquist plots, respectively.

The binding strength between the target analyte and the recognition sites of the MIP sensor was evaluated through specific binding analysis. The binding affinity of E2 molecules to MIP-E2 sensors was analysed by fitting the plot of the difference of SWV peak currents (Δ*I*_BA_), against E2 concentrations using the heterogeneous Langmuir–Freundlich (LF) isotherm model as expressed in eqn (2) (Fig. 5g). The LF binding isotherm was used because electropolymerized MIP layers typically exhibit a finite number of heterogeneous binding sites formed during polymerization. This behavior deviates from ideal homogeneous adsorption and is therefore well described by the LF model, which has been widely applied to MIP-based electrochemical sensors.^43,63,64^2
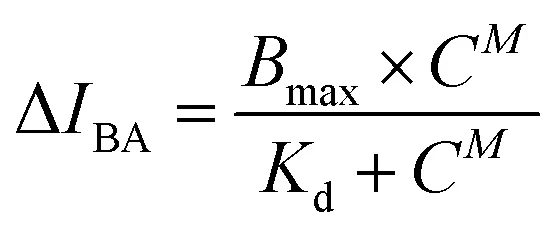
Δ*I*_BA_ is the difference in the SWV peak current. *M* is a heterogeneous parameter, a number between 0 and 1 that refers to the heterogeneity of the binding sites. *K*_d_ is the dissociation constant related to the binding strength between E2 molecules and the imprinted cavities in the MIP-E2 sensor. *B*_max_ is the maximum adsorption capacity.

By fitting the experimental data with the LF isotherm model, the *M*, *K*_d_, and *B*_max_ were obtained as 0.38, 11.2 nM, and 15.03 µA, respectively. The small *M* value indicated a heterogeneous sensor surface, which broadens the dynamic range of concentration detection, making it advantageous for practical sensing applications, while the lower *K*_d_ suggested strong binding (high affinity) between E2 target molecules and MIP-E2 sensors.

The specific binding isotherm (*S*_b_) was obtained by subtracting the different peak current (Δ*I*) of NIP sensors from MIP-E2 sensors and normalizing by the sensing area to eliminate non-specific binding. As shown in Fig. 5h, the *S*_b_ curve exhibited a steep slope and a measurable current response of 0.86 µA at the lowest tested E2 concentration (0.5 nM), indicating that the MIP-E2 sensor possessed high sensitivity and capability of low-level detection of E2.

### 3.5 Selectivity studies

The selectivity of the MIP-E2 sensor was assessed by comparing the binding behaviour of E2 with structurally analogous molecules, including estrone (E1) and estriol (E3) (Fig. 6). The results showed that the SWV current difference (Δ*I*) generated by binding of E2 (1.5–9 µA) was higher than that of E1 and E3 (1–2.5 µA) over the tested concentration range (0.5–500 nM). The negligible measurable change in current response for E1 and E3 demonstrated insignificant cross-reactivity of MIP-E2 sensors with structurally analogous molecules. The presence of a hydroxyl group in the E2 molecule at the C-17 position imparts higher selectivity toward E2 than toward E1 and E3 that contain a ketone functional group at the C-16 position and an additional hydroxyl group at the C-16 position, respectively. The positional differences of these functional groups on the aromatic rings significantly influenced the binding efficiency of E2 to the MIP-E2 sensors. These results indicated that the MIP-E2 sensors exhibited high selectivity with a broad dynamic linear range and excellent sensitivity for E2 detection, showing the potential for reliable sensing in practical applications.

**Fig. 6:**
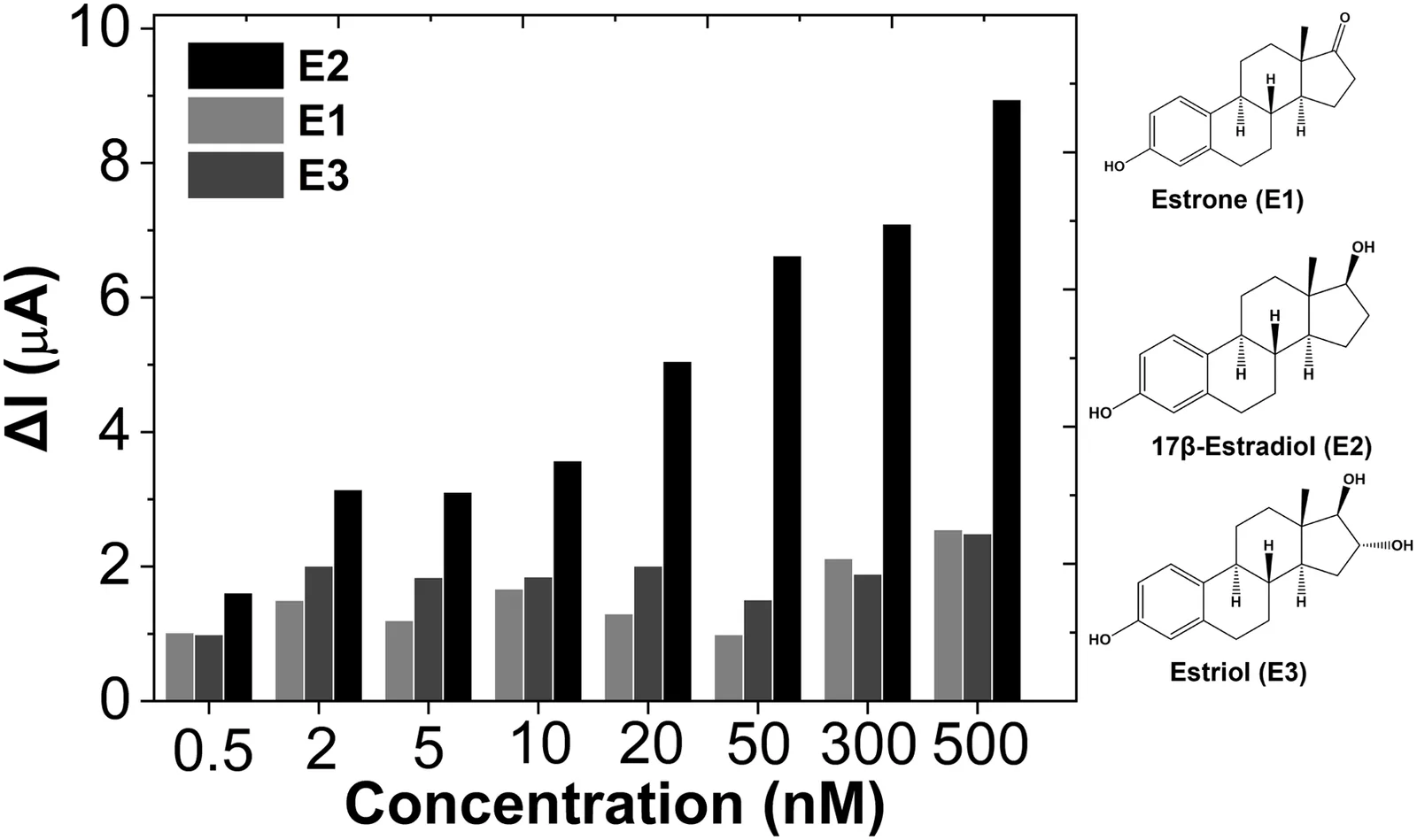
SWV current difference (Δ*I*) against various concentrations of structurally analogous molecules, including E1, E2, and E3, measured utilizing MIP-E2 sensors. All measurements were performed in 0.1 M KCl solution containing 5 mM redox probes. Δ*I* corresponded to the difference between the current recorded after template removal (*I*_0_) and the current recorded after incubation in a PBS solution containing a specific concentration of E2 (*I*_t_).

### 3.6 Detection of E2 in wastewater samples

To assess the practical applicability of the sensors, wastewater was employed as an incubating medium, as it is a complex medium consisting of various organic and inorganic constituents.^65^ The wastewater was collected from Aquafin Hasselt and was filtered to remove large debris by passing through a Whatman filter paper (pore size 2 µm). This enabled the investigation of the sensor's selective binding under realistic and interference-rich conditions. Subsequently, the MIP-E2 sensor was incubated in wastewater containing different concentrations of E2 (0.5–500 nM) for 10 min, followed by SWV and EIS measurements. The SWV current response of the MIP-E2 sensor at ∼0.23 V *vs.* Ag/AgCl decreased as the E2 concentration increased, while the response of the NIP sensors exhibited negligible change (Fig. 7a and b). These results demonstrated specific rebinding of E2 molecules to the recognition sites in the MIP matrix, however, no significant binding was observed in the NIP matrix. Besides, the MIP-E2 sensors exhibited higher sensitivity than that of NIP sensors for E2 detection in wastewater, as described by the higher slope of the linear equations Δ*I* = 2.44 [*C*] + 2.77 (*R*^2^ = 0.87) of MIP-E2 sensors, compared to Δ*I* = 1.36[*C*] + 0.42 (*R*^2^ = 0.86) of NIP sensors. The EIS results agreed with SWV data, showing that the impedance increased with E2 concentration for the MIP-E2 sensors, whereas the NIP sensors exhibited only negligible changes in impedance (Fig. 7d and e). Moreover, the calibration curves plotted between real impedance (*Z*′) and E2 concentration confirmed a significant linear increase in *Z*′ with E2 concentration for MIP-E2 sensors, whereas a slight increase in *Z*′ was observed for NIP sensors (Fig. 7f). These results corroborated higher sensitivity and specificity toward E2 of MIP-E2 sensors, compared to NIP sensors, as expressed by the slope in the linear equations of *Z*′ = 218.13[*C*] + 600.22 (*R*^2^ = 0.98) and *Z*′ = 83[*C*] + 387.43 (*R*^2^ = 0.88) for MIP-E2 and NIP sensors, respectively.

**Fig. 7:**
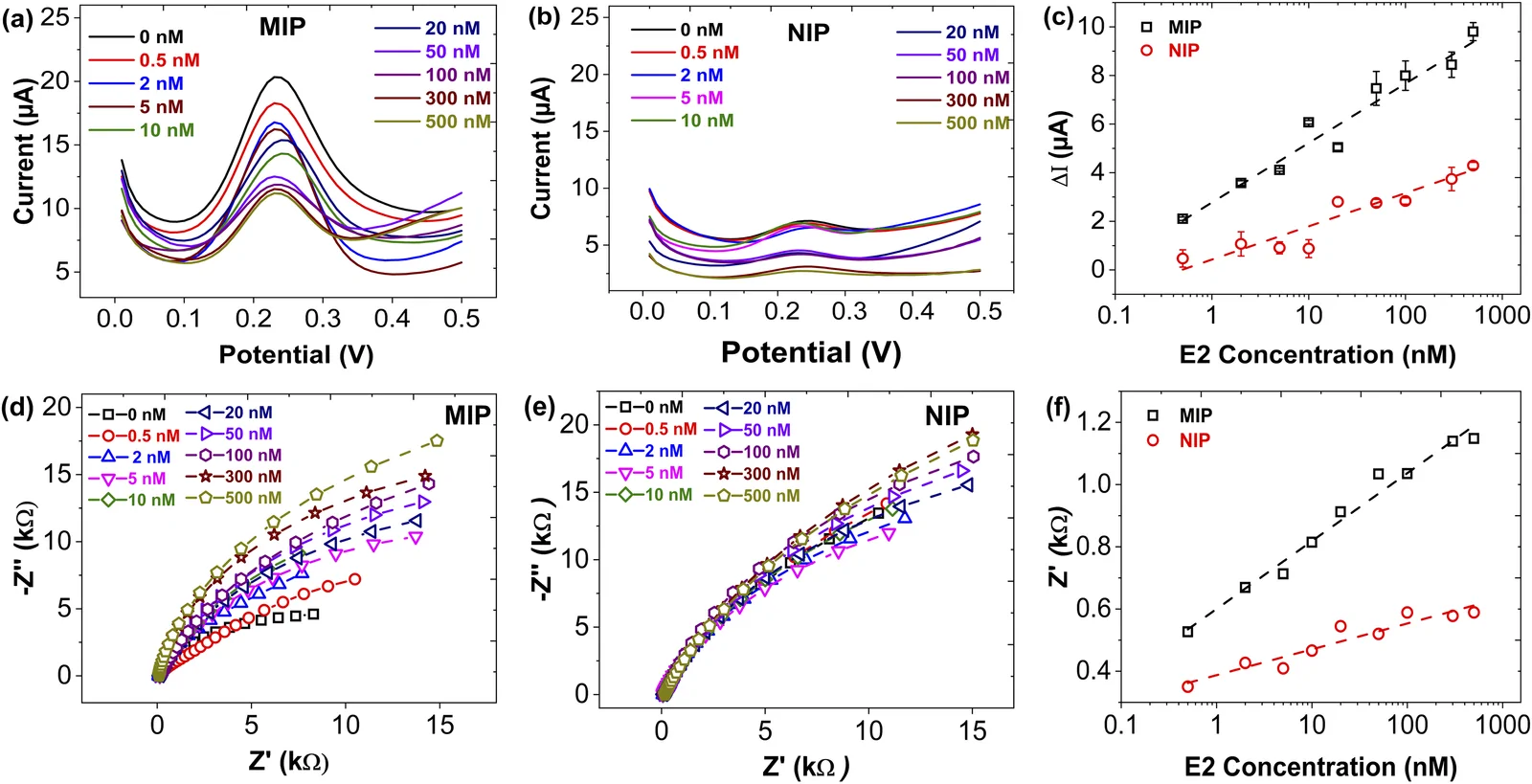
Square wave voltammograms for (a) MIP-E2 and (b) NIP sensors after incubated with different concentrations of E2 (0–500 nM in wastewater). (c) Calibration curves for MIP-E2 and NIP sensors, *n* = 3. Nyquist plots for (d) MIP-E2 and (e) NIP sensors after incubated with different concentrations of E2 (0–500 nM in wastewater). (f) Calibration curves plotted with real impedance (*Z*′) against E2 concentration (0–500 nM in PBS) for MIP-E2 and NIP sensors. All the measurements are recorded in a 0.1 M KCl solution containing 5 mM redox probes.

Compared with other MIP-based sensors for E2 detection, the MIP-E2 sensor showed a broad linear detection range across a wide concentration window (from 0.5 to 500 nM), while maintaining a low detection limit of 0.41 nM (Table S2).

## 4. Conclusions

This work developed an MIP-based electrochemical sensor for E2 detection by upcycling rPET films into conductive electrode substrates, followed by electropolymerization of o-PD in the presence of E2 templates and gold nanoparticles (AuNPs). The AuNPs in the MIP matrix significantly enhanced the conductivity and facilitated the charge transfer, as demonstrated by improved sensitivity of the sensor toward E2 detection. Key parameters for MIP fabrication were optimized to achieve maximum sensing performance (current response), including the number of CV scan cycles, monomer–template ratio, pH of electrolyte, washing time, and eluents used to remove the E2 template. The MIP-E2 sensors exhibited high sensitivity and specificity over a linear detection range of E2 concentration between 0.5 and 500 nM in PBS with a LOD of 0.18 nM in buffer solution. The binding of E2 target analyte to recognition cavities within the MIP matrix suppressed electron transfer between redox probes and electrode surface, resulting in a concentration-dependent decrease of current response. Owing to the specific binding sites in the MIP matrix, the MIP-E2 sensors showed high affinity for rebinding of E2, with a dissociation constant (*K*_d_) of 11.2 nM. Moreover, the sensors demonstrated excellent selectivity of E2 compared with its structural analogues (E1 and E3). The sensing performance in wastewater without time-consuming pretreatment steps and with a LOD of 0.41 nM, highlights that the sensors remain functional in realistic environments containing complex constituents in wastewater. Overall, in this work, the fabrication of MIP sensors *via* upcycling recycled PET plastic was demonstrated by employing E2 as a model template/analyte. The proposed approach can be extended to other recycled plastics and adapted to sensing a broad spectrum of wastewater pollutants, including estrogens, pesticides, organic contaminants, and pharmaceutical residues. Future work will focus on validating sensor performance within analyte-specific concentration ranges through complementary analytical techniques, supporting its translation into practical environmental monitoring applications. Furthermore, the MIP-E2 sensor developed by rPET electrode offers lighter weight compared to bulk rigid metal electrodes, offering a significant practical advantage, particularly for applications in portable and wearable devices. Such sensors also offer a sustainable and value-added solution, which aligns with the circular economy principle by valorizing discarded plastics into functional devices.

## Author contributions

Muhammad Faran Tahir: conceptualization, data acquisition, formal analysis, data curation, visualization, writing – original draft. Tiwa Yimyai: data analysis, scientific discussion, writing – review & editing. Jan D'Haen: SEM images acquisition, data analysis, writing – review & editing. Ronald Thoelen: validation, scientific discussions, writing – review & editing. Anitha Ethirajan: conceptualization, validation, resources, writing, review & editing, supervision, project administration, funding acquisition.

## Conflicts of interest

The authors declare no conflict of interest.

## Supplementary Material

RA-OLF-D6RA09742A-s001

## Data Availability

All the data supporting the findings of this article are provided in the supplementary information (SI). Supplementary information: optimal formulation for MIP and NIP electrode fabrication (Table S1); photographs illustrating the physical appearance of recycled PET substrates and fabricated MIP sensor (Fig. S1); optimization of electropolymerization parameters (Fig. S2–S7); electrochemical characterization and comparison of MIP sensor performance in the presence and absence of gold nanoparticles (Fig. S8); surface morphology and characterization using AFM, SEM, and contact angle measurements (Fig. S9–S11); equivalent circuit modeling and EIS analysis (Fig. S12 and S13); and a comparison of electropolymerized MIP-based sensors for E2 detection in water samples (Table S2). See DOI: https://doi.org/10.1039/d6ra09742a.
