# Supplementary material for: Upcycling recycled PET into a conductive electrode for a molecularly imprinted polymer based electrochemical sensor for 17β-estradiol detection
Source: RSC Adv. 2026 Nov 10. Online ahead of print. doi: 10.1039/d6ra09742a (PMC13749436; doi:10.1039/d6ra09742a)
Supplement: Supplementary file 1 [file RA-OLF-D6RA09742A-s001.pdf]

Supplementary Information (SI) for RSC Advances.
This journal is © The Royal Society of Chemistry

Supplementary Information

Upcycling recycled PET into a conductive electrode for molecularly imprinted polymer based electrochemical sensor for 17β-estradiol detection

Muhammad Faran Tahir, [ab] Tiwa Yimyai, [ab] Jan D'Haen, [ab] Ronald Thoelen, [ab] Anitha Ethirajan [*ab]

a. UHasselt, Institute for Materials Research (IUMAT), Martelarenlaan 42, Hasselt B-3500, Belgium.

b. imec, IUMAT, Wetenschapspark 1, Diepenbeek B-3590, Belgium.

***Corresponding author:** anitha.ethirajan@uhasselt.be

Preparation of MIP/NIP sensor

Table S1. Optimal formulation for the synthesis of the MIP and NIP electrodes.

Chemical	Function	Solvent	MIP	NIP
o-PD	Monomer	0.1 M acetate buffer solution, pH 4	5 mM	5 mM
$HAuCl_4$	Catalyst/conductive nanofiller	0.1 M KCl	0.1 mM	0.1 mM
E2	Template	Ethanol	0.5 mM	-

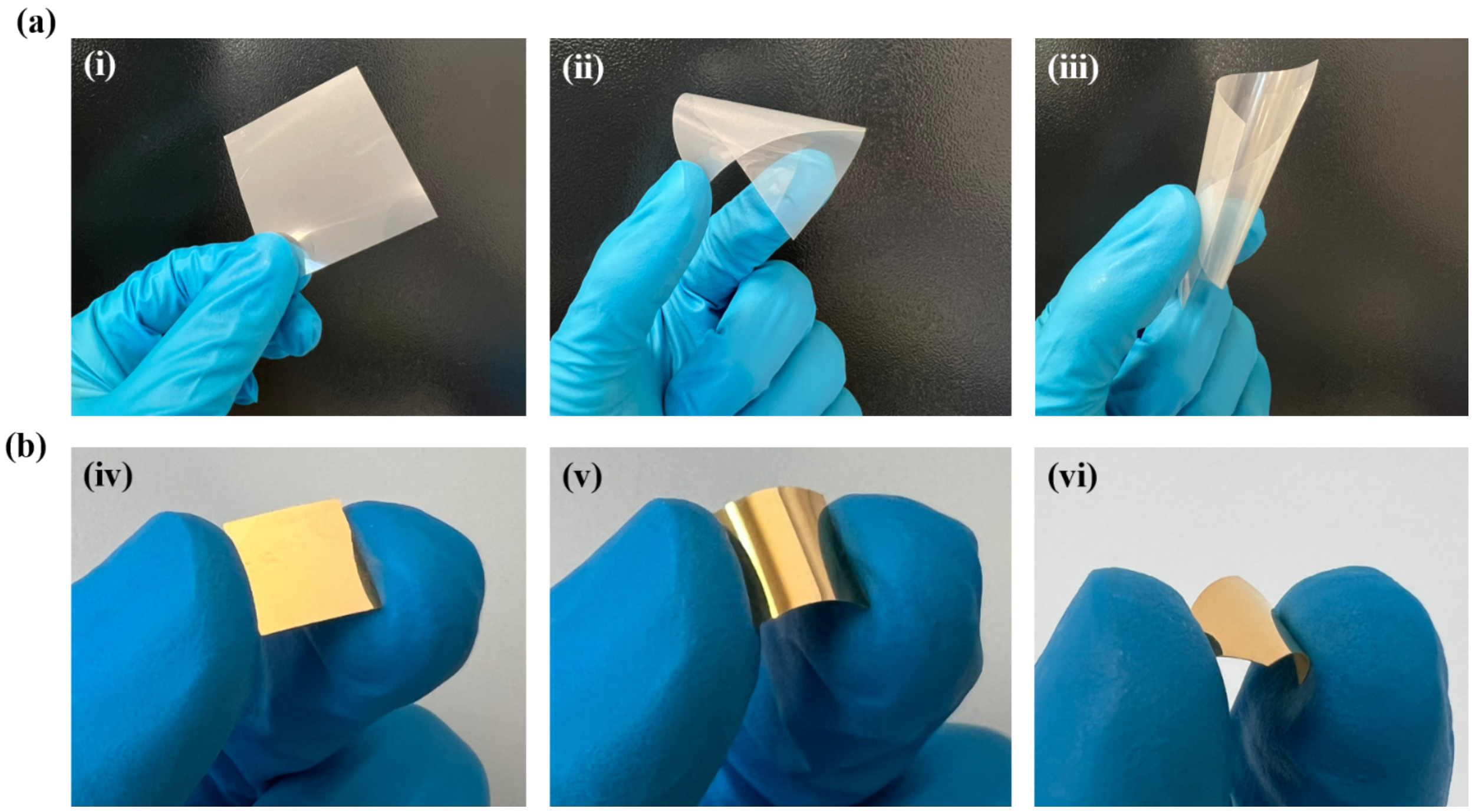

Figure S1. (a) Photographs showing the physical appearance of the flexible recycled PET film (5 cm x 5 cm) before gold deposition: (i) flat, (ii) bent, and (iii) rolled configurations. (b) Photographs representing the physical appearance of the electropolymerized MIP on gold-coated flexible rPET substrate (MIP-E2 sensor, 1 cm x 1 cm): (iv) flat, and (v−vi) bent configurations, shown from two different viewing angles.

Optimization of parameters for MIP fabrication via electropolymerization

The key parameters, including electropolymerization cycles, the effect of pH of the acetate buffer solution, monomer-to-template ratio, eluents to remove E2 molecules, washing time, and incubation time, were optimized for the fabrication of the MIP-E2 sensors. In the following section (Figures S2−S7), the optimization of each parameter was discussed in detail, and each parameter was plotted against the change in current response (Δ*I*). Δ*I* can be defined as the difference between the current recorded after template removal (I_o) and the current recorded after incubation in a 100 nM E2 solution in PBS (I_t).

CV scan cycles

Different MIP layers were prepared using various CV cycles of electropolymerization (15, 20, 25, 30, and 35) by keeping the scan rate at 50 mVs^{-1}, pH of the solution at 4 and a monomer-template ratio of 10. As shown in Figure S2, the maximum Δ*I* of MIP sensors is achieved with 25 cycles of polymerization.

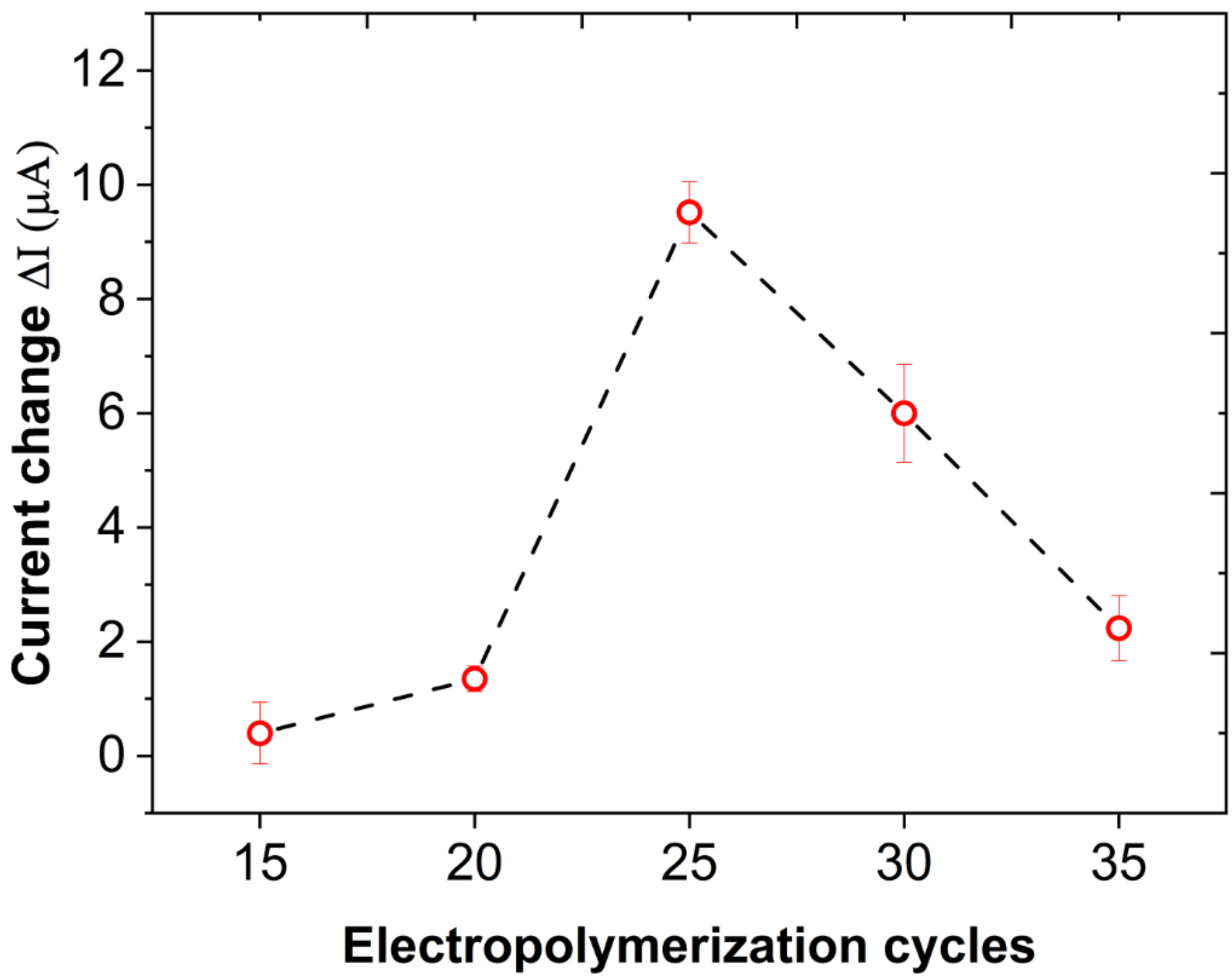

Figure S2. Current changes (ΔI) of MIP-E2 sensor as a function of CV cycles of electropolymerization. All measurements were performed using SWV by applying a potential from

0 to 0.5 V vs Ag/AgCl, a pulse size of 25 mV, and at a frequency of 10 Hz with a 0.1 M KCl solution containing 5 mM redox probes.

Effect of pH

The pH of the medium used for the electrodeposition plays an important role in the performance of the MIP electrode. The MIP layers were deposited using the optimal formulation (Table S1) at pH 4, employing 25 CV cycles of polymerization and a monomer-template ratio of 10 (*confer vide infra*). The highest Δ*I* was achieved with a solution at pH 4 using 25 CV cycles (Figure S3).

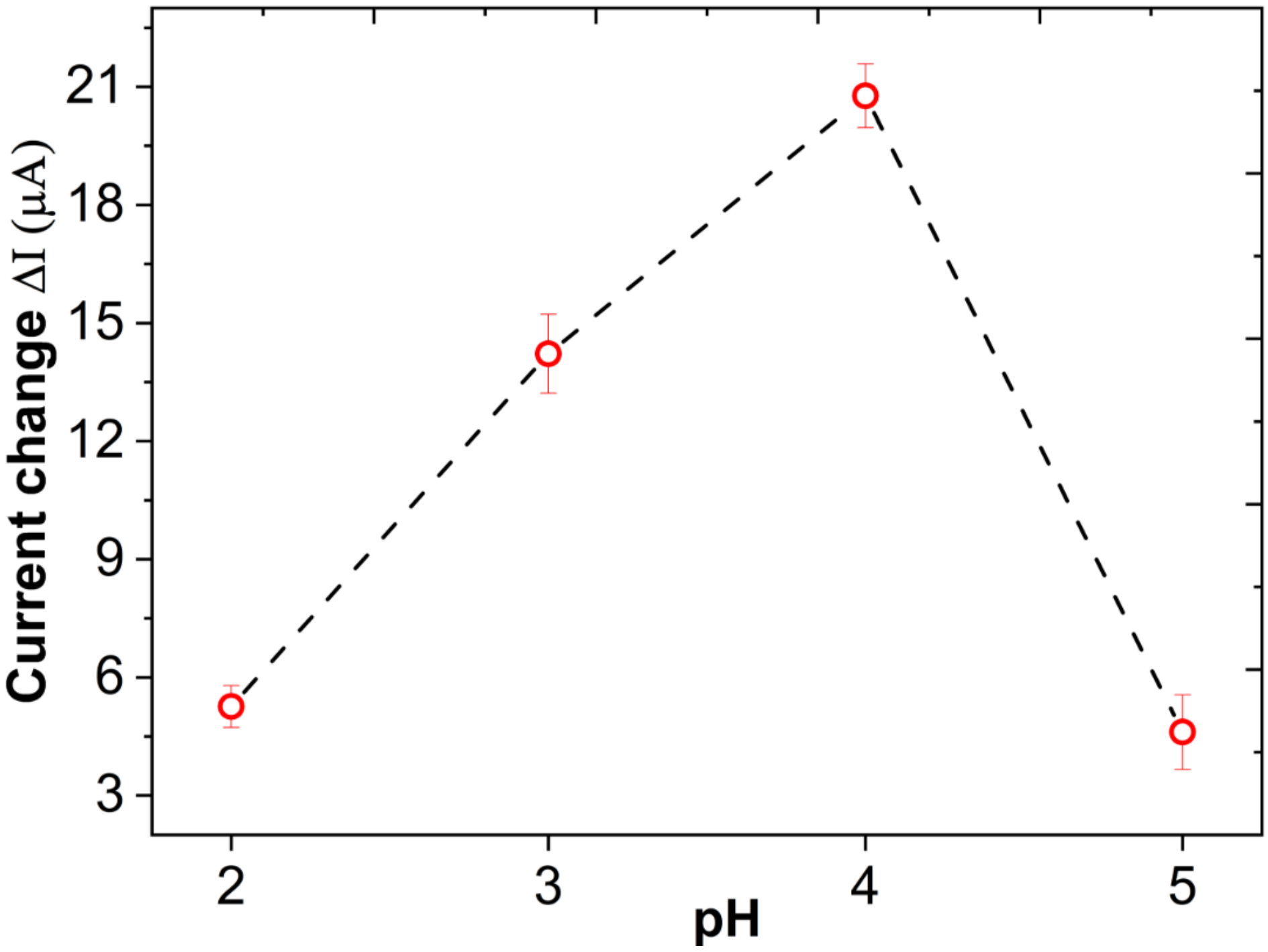

Figure S3. Effect of the pH of the electrolyte for electropolymerization of *o*-PD for the MIP electrode. All measurements were performed using SWV by applying a potential from 0 to 0.5 V vs Ag/AgCl, a pulse size of 25 mV, and at a frequency of 10 Hz with a 0.1 M KCl solution containing 5 mM redox probes.

Monomer-template ratio

The molar ratio between monomer (*o*-PD) and template (E2) played a crucial role in defining the performance of the MIP electrode during the electropolymerization process. The MIP layers were deposited using the optimal formulation (Table S1) at pH 4 and employing 25 CV cycles of polymerization. The highest Δ*I* was achieved with the monomer-template ratio of 10 (5 mM *o*-PD: 0.5 mM E2 template).

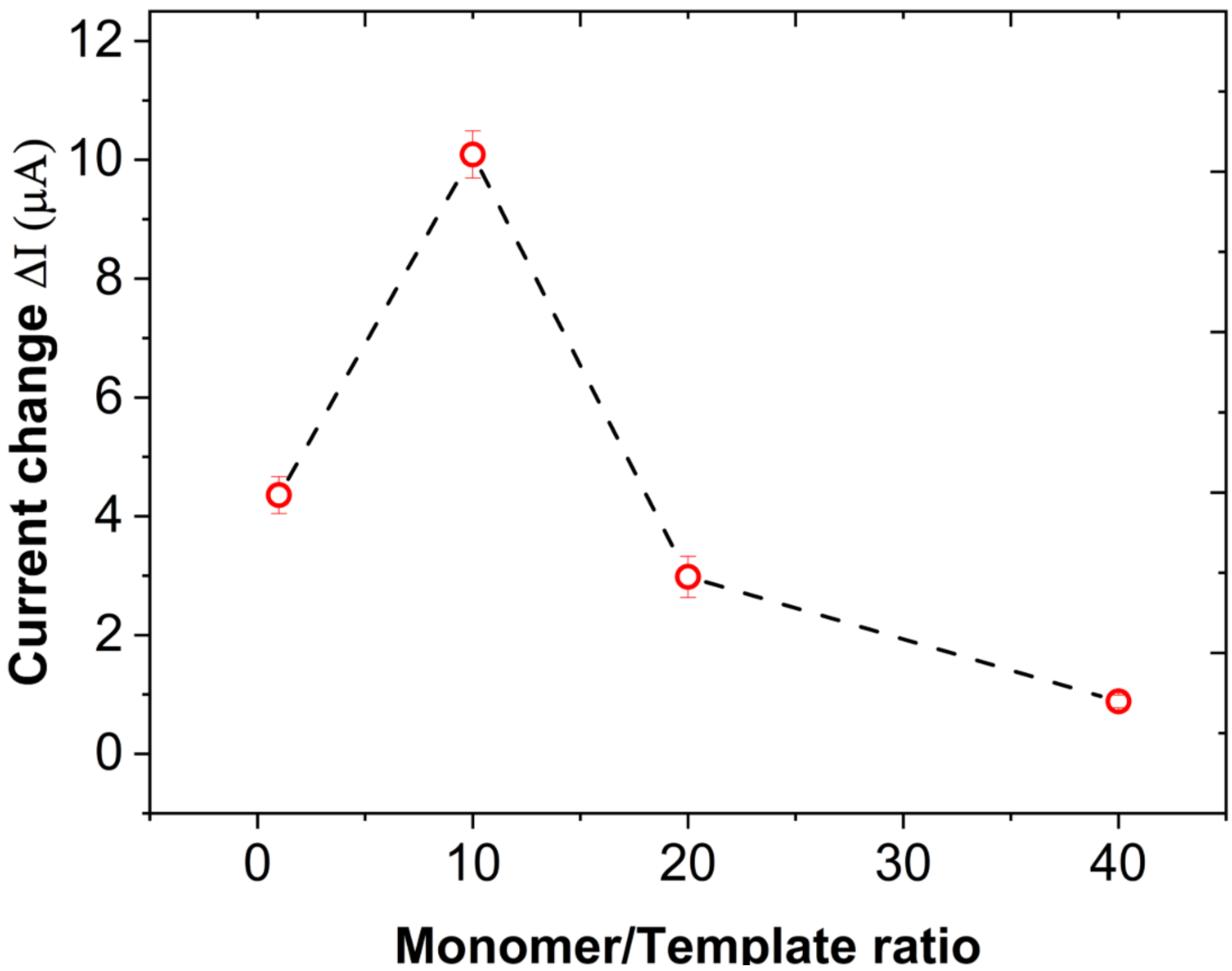

Figure S4. Effect of monomer-template ratio for electropolymerization. All the measurements were performed using SWV by applying a potential from 0 to 0.5 V vs Ag/AgCl, a pulse size of 25 mV, and at a frequency of 10 Hz in a 0.1 M KCl solution containing 5 mM redox probes.

Washing time

The MIP layers were deposited using the optimal formulation (Table S1) at pH 4, employing 25 CV cycles of polymerization and a fixed monomer-template ratio of 10. The SWV current response of the MIP-E2 sensor after different washing intervals (2, 5, 10, 15, 20, and 25 min) was studied. The maximum Δ*I* achieved was after 10 min of washing, suggesting efficiency in removing the E2 template molecules from the MIP films.

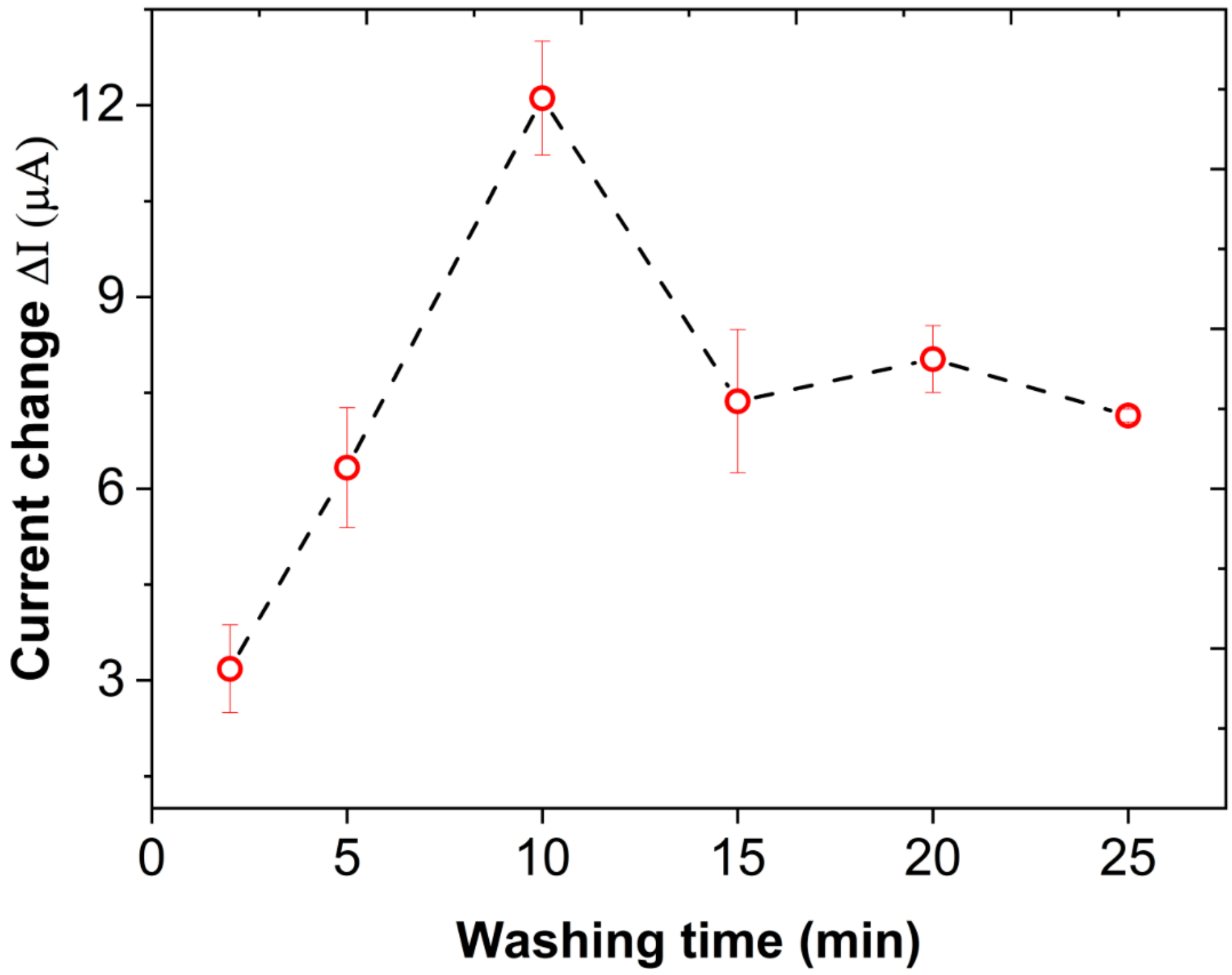

Figure S5. Current change (ΔI) of MIP-E2 sensors obtained at different washing times. All the measurements were performed using SWV by applying a potential from 0 to 0.5 V vs Ag/AgCl, a pulse size of 25 mV, and at a frequency of 10 Hz in a 0.1 M KCl solution containing 5 mM redox probes.

Eluents for template removal

Different eluents, including phosphate-buffered saline (PBS), acetic acid, methanol (1:19), ethanol, and acetonitrile, were investigated for template removal from the MIP layer, which was deposited using the optimal formulation (Table S1) at pH 4, employing 25 CV cycles of polymerization and a fixed monomer-template ratio of 10. The washing time was fixed at 10 minutes (see the previous section for the optimization of washing time). As shown in Figure S6, the MIP after washing with acetonitrile showed the highest current response, compared to other washing solvents.

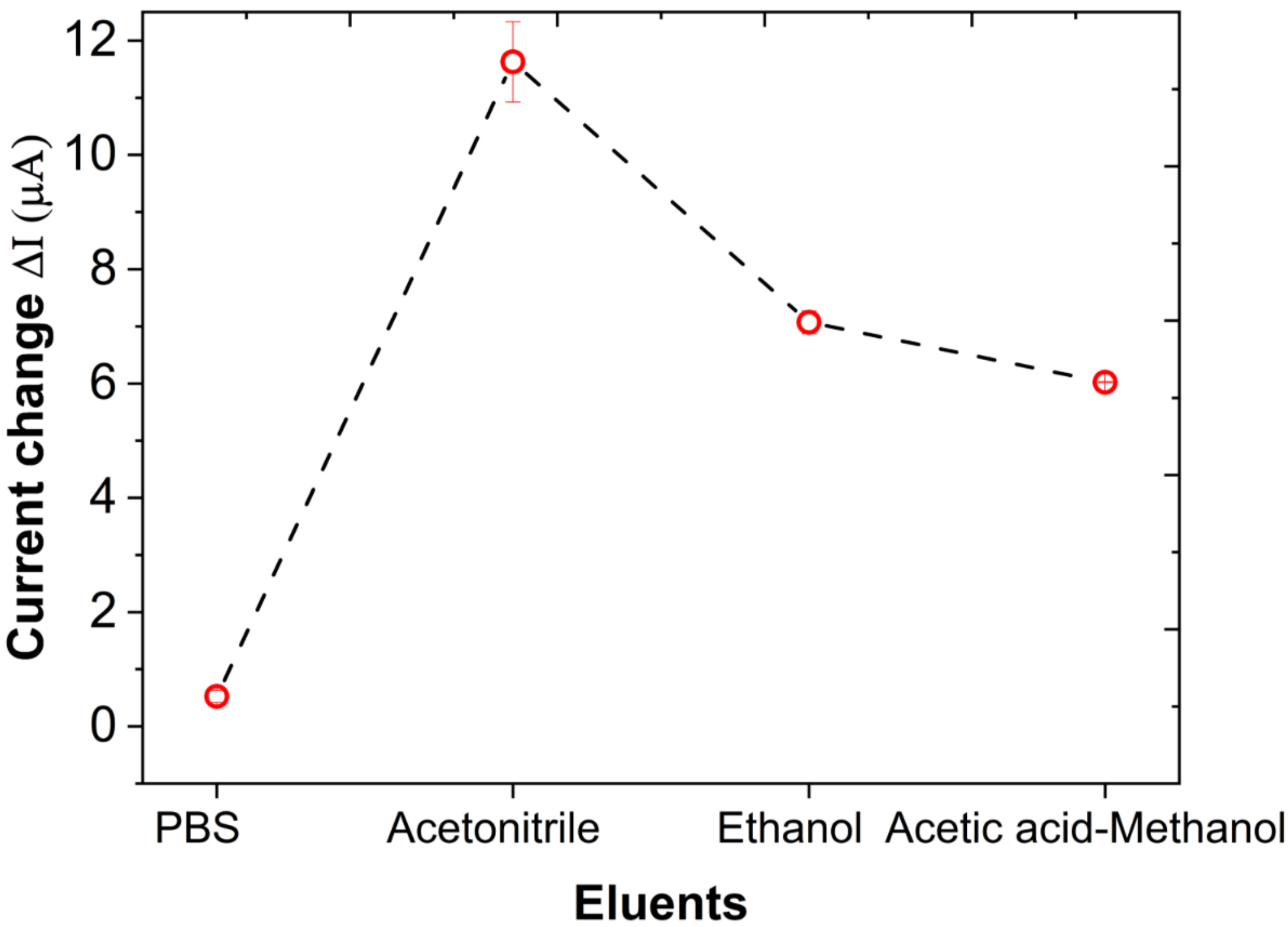

Figure S6. Current change (Δ*I*) of the MIP-E2 sensors using different eluents for 10 min template removal. All the measurements were performed using SWV by applying a potential from 0 to 0.5 V vs Ag/AgCl, a pulse size of 25 mV, and at a frequency of 10 Hz in a 0.1 M KCl solution containing 5 mM redox probes.

Incubation time

A MIP layer was deposited using the optimal formulation (Table S1) at pH 4, employing 25 CV cycles of polymerization and a monomer-to-template ratio of 10. The MIP layer was washed for 10 min in acetonitrile solution. Then, the incubation study was performed by immersing the MIP-E2 sensor in PBS containing 100 nM E2. As shown in Figure S7, an incubation time of 10 min was selected for subsequent measurements.

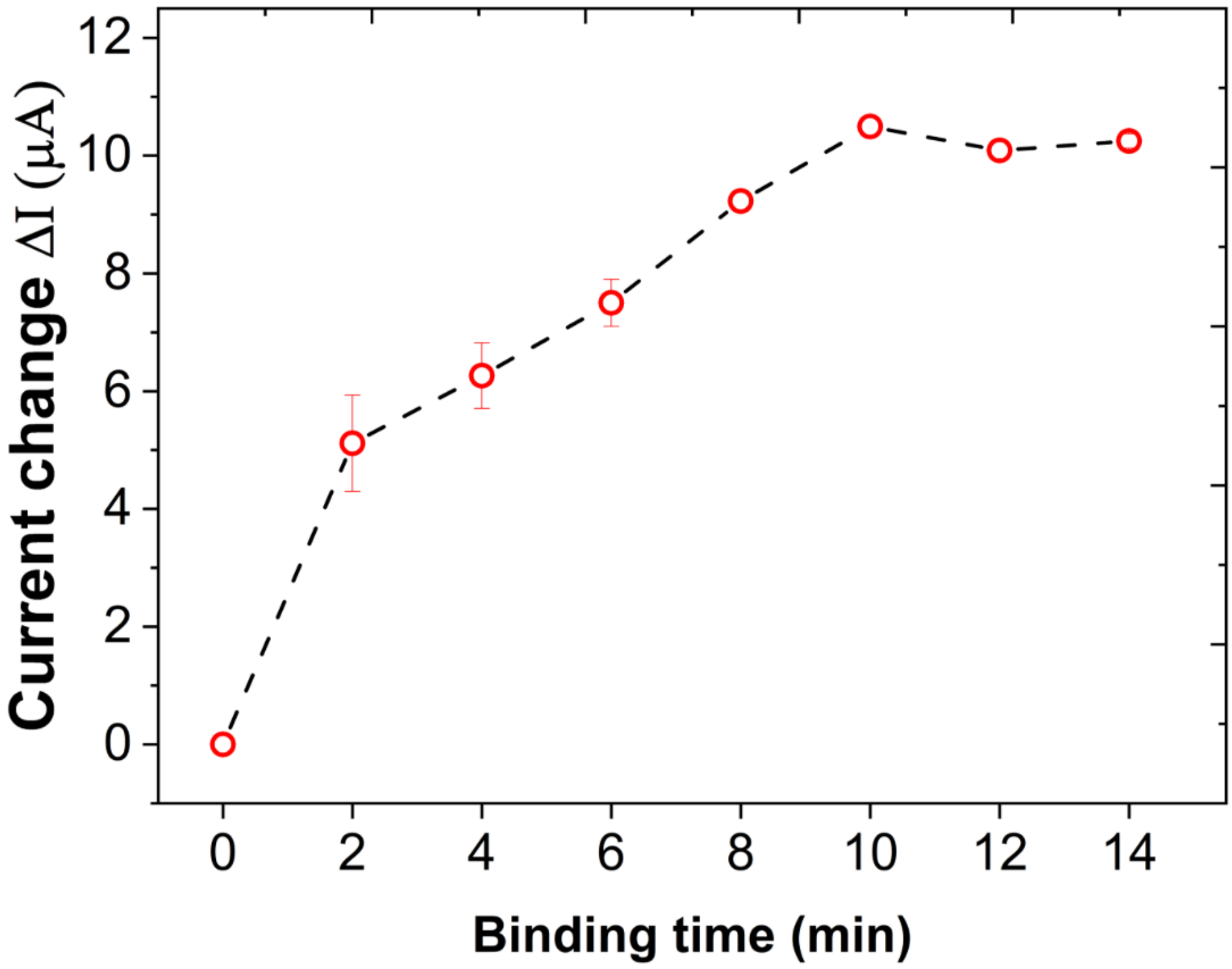

Figure S7. Effect of incubation time of E2 target molecule for MIP-E2 sensors. All the measurements were performed using SWV by applying a potential from 0 to 0.5 V vs Ag/AgCl, a pulse size of 25 mV, and at a frequency of 10 Hz in a 0.1 M KCl solution containing 5 mM redox probes.

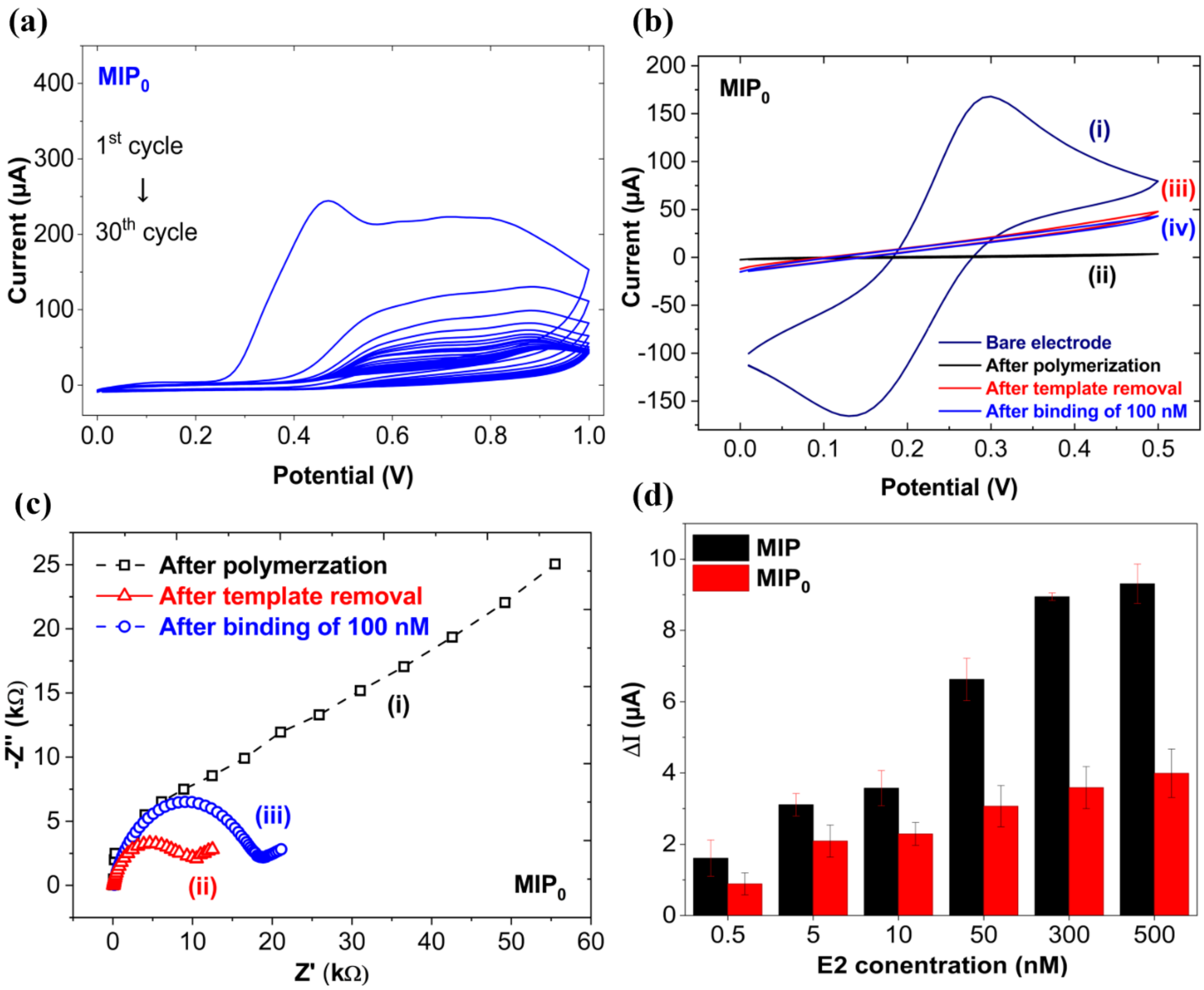

Figure S8. (a) Representative cyclic voltammogram during electropolymerization for MIP$_0$ in a solution containing 5 mM *o*-PD in 0.1 M acetate buffer solution (pH 4) and 0.5 mM E2 template molecule in ethanol. (b) Cyclic voltammograms and (c) Nyquist plots obtained by electrochemical impedance spectroscopy (EIS) for (i) bare electrode, (ii) after electropolymerization, (iii) after template removal, and (iv) after rebinding of E2 (100 nM in PBS). (d) Current response of MIP-E2 and MIP$_0$-E2 sensors after incubation in various E2 concentrations in PBS (n=3). The measurements for results shown in Figure S8(b−d) were performed in a 0.1 M KCl solution containing 5 mM redox probes.

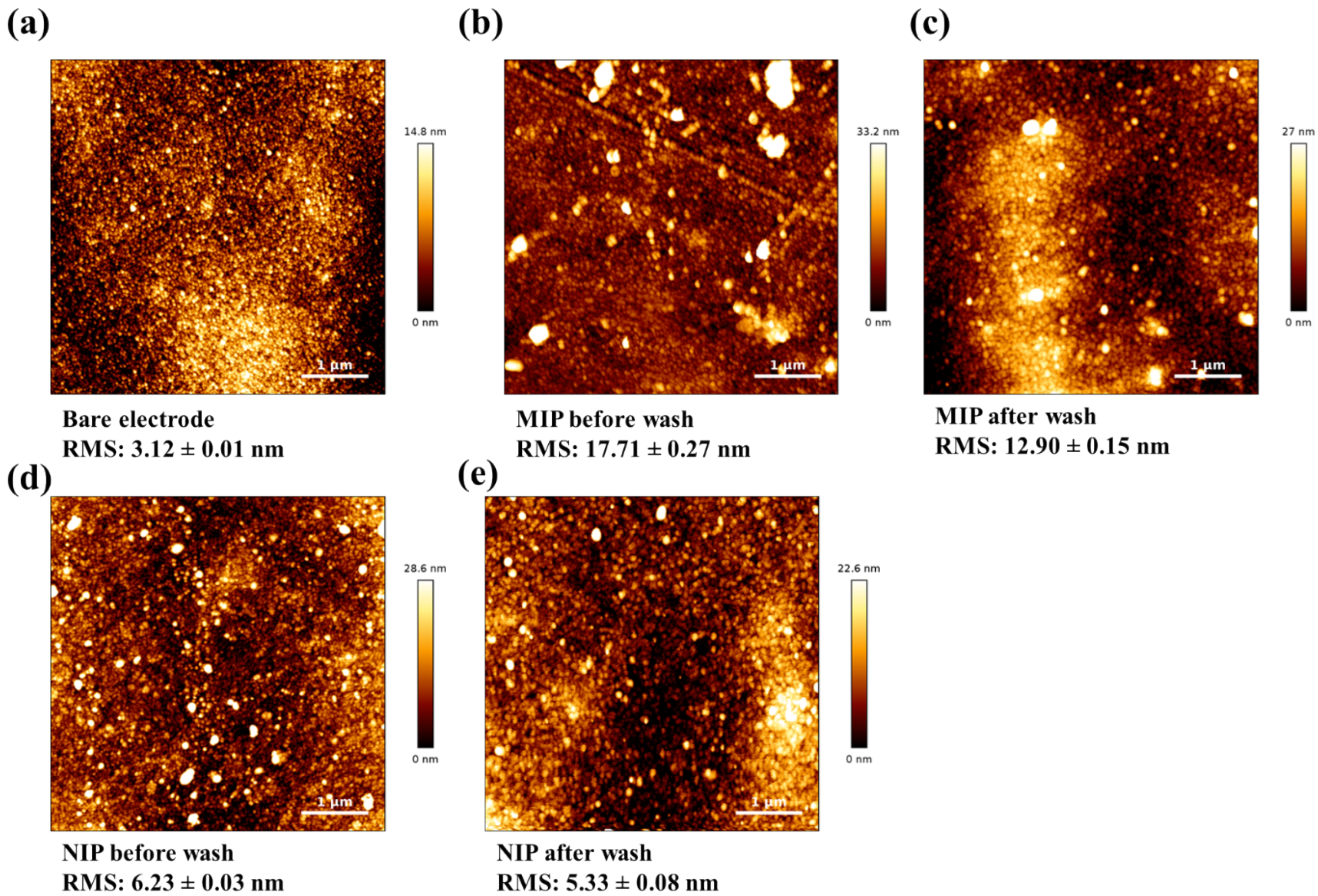

Figure S9. Representative AFM images of (a) bare electrode; MIP electrode (b) before and (c) after template removal; NIP electrode (d) before and (e) after template removal. The root mean square (RMS) roughness of the respective electrodes was averaged from three measurements.

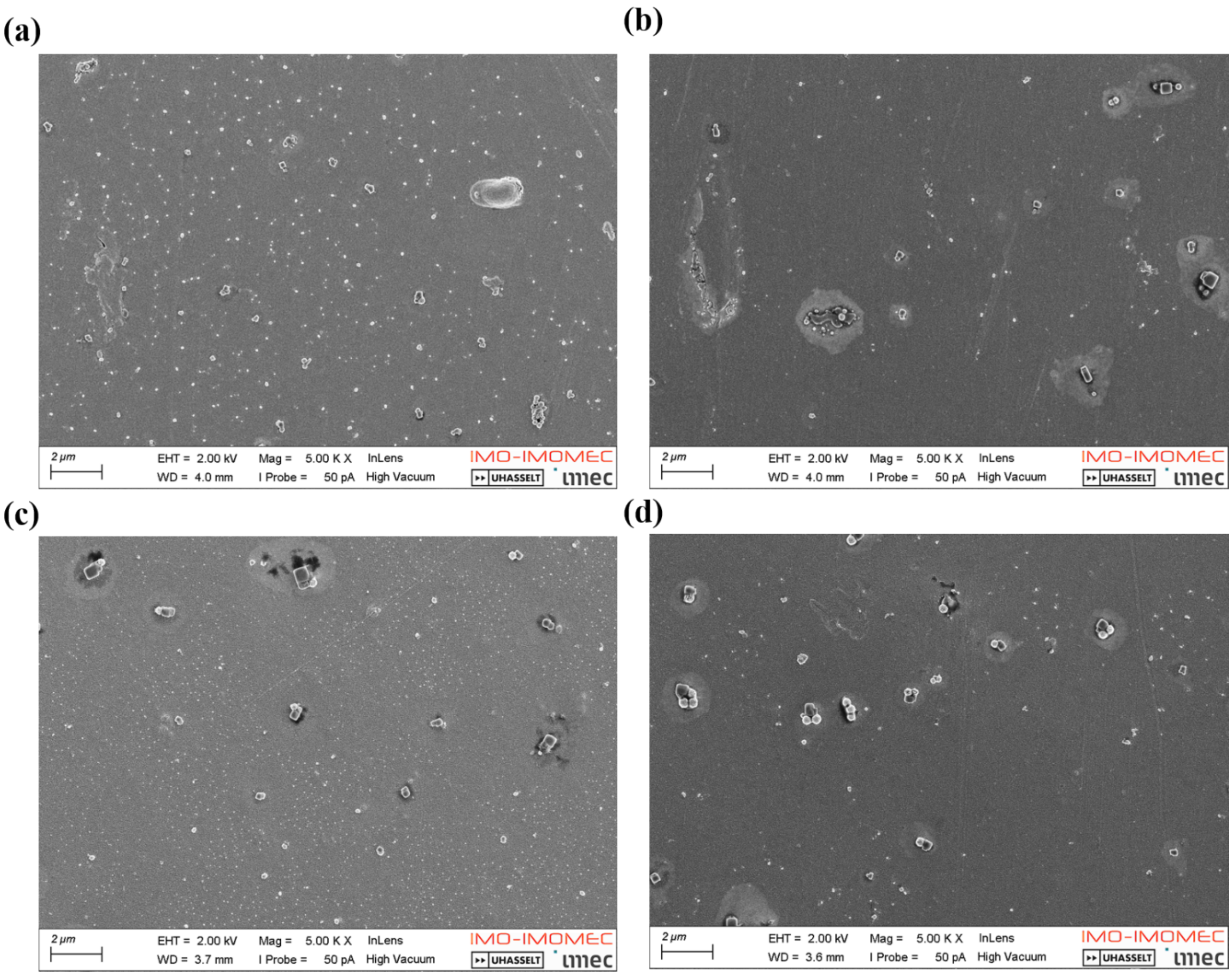

Figure S10. Representative SEM images of the MIP-E2 sensor (a) before and (b) after washing, and the NIP sensor (c) before and (d) after washing.

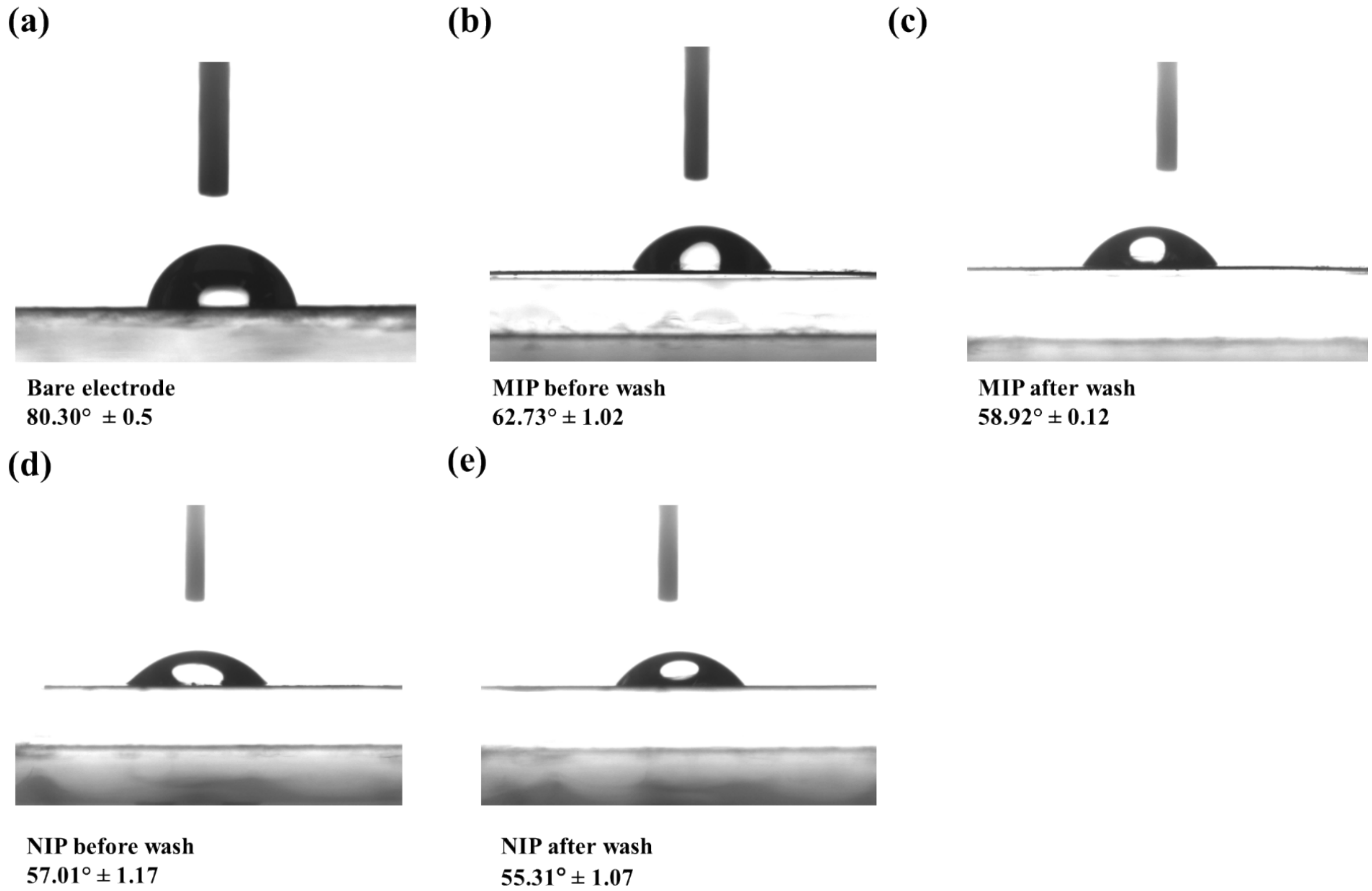

Figure S11. Contact angle images of a water droplet on (a) bare gold; MIP (b) before and (c) after template removal; NIP (d) before and (e) after template removal. The contact angle values were obtained from three measurements.

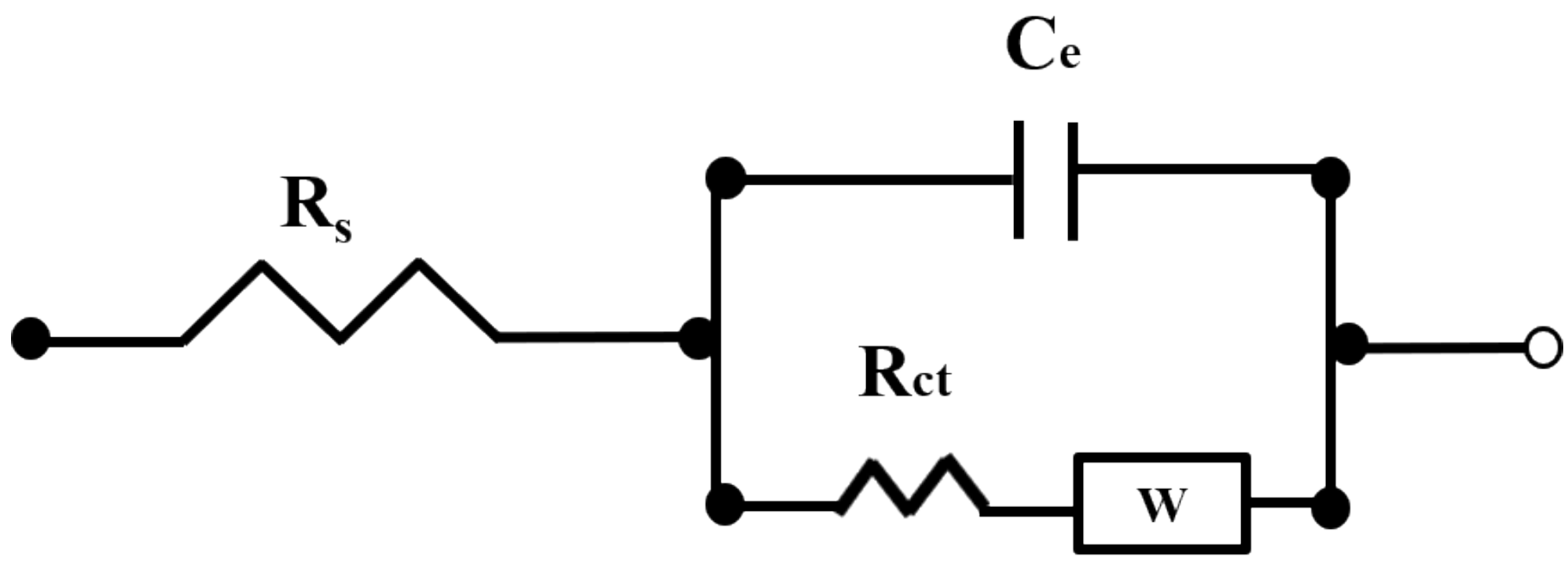

Figure S12. Equivalent circuit used to fit the EIS response of the MIP-E2 sensor. *R*s represents the ohmic electrolyte/series resistance, R_{ct} the effective interfacial charge-transfer resistance at the MIP-modified electrode, C_e the effective interfacial capacitance, and *W* the Warburg diffusion element associated with mass transport of the redox species/electrolyte.

(a)

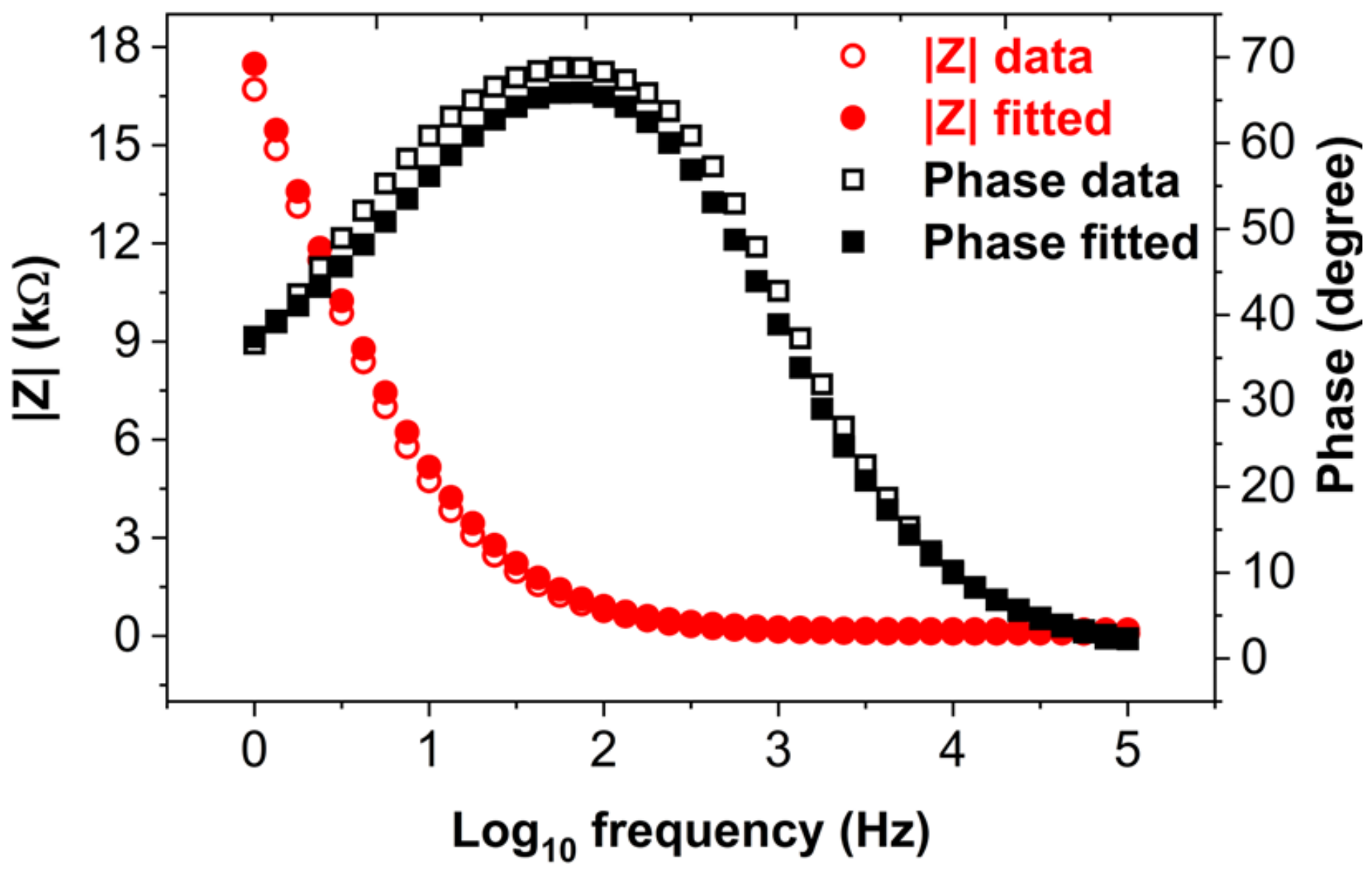

(b)

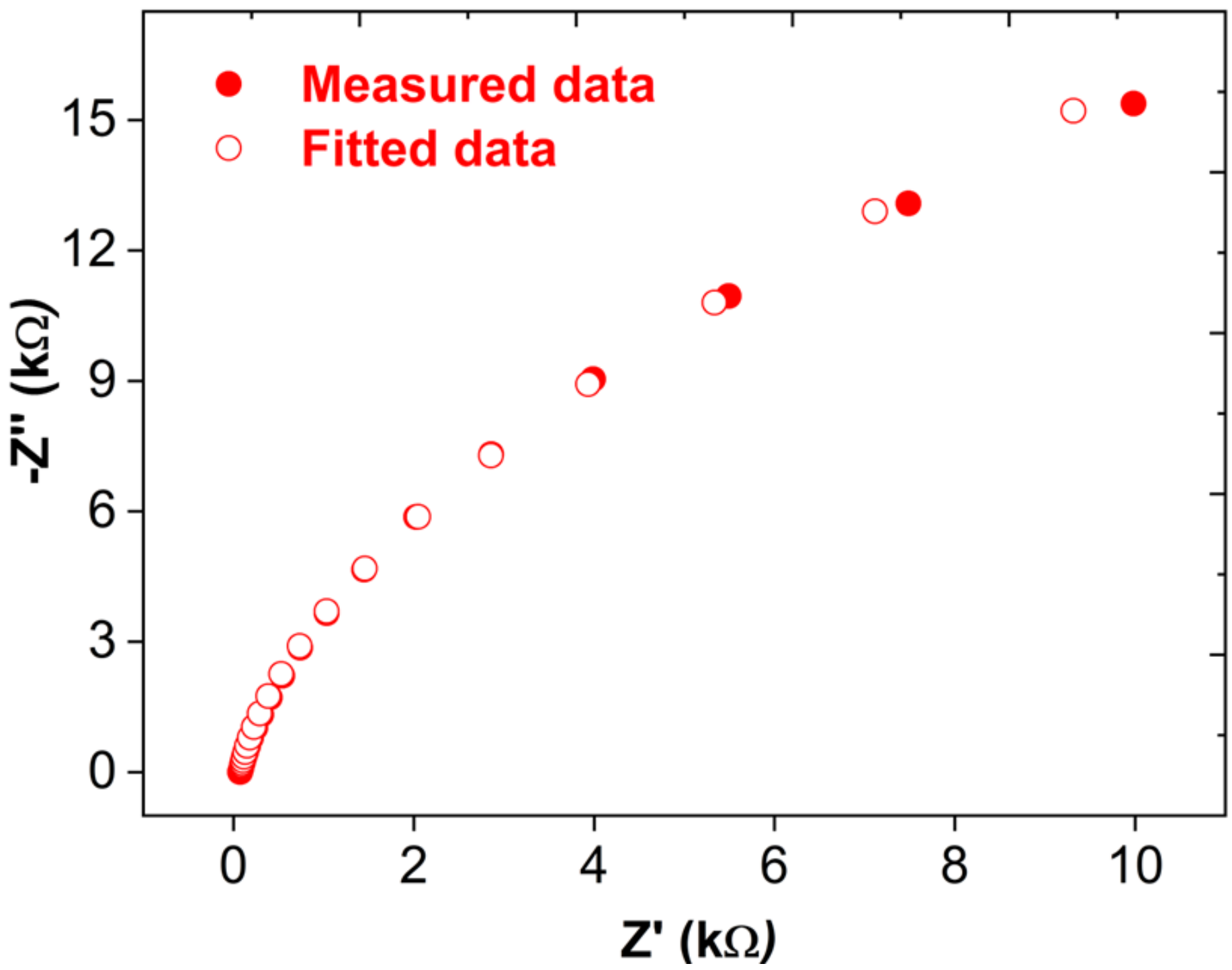

Figure S13. (a) Bode plots and (b) Nyquist plots of MIP-E2 sensor incubated in E2 solution (100 nM in PBS) obtained from EIS by applying a potential of 10 mV vs Ag/AgCl in a frequency range from 1 Hz to 100 kHz. All the measurements were performed in a 0.1 M KCl solution containing 5 mM redox probes.

Table S2. Comparison of electropolymerized MIP-based sensors for the detection of E2 in water samples.

Functional monomer	Working electrode	Readout	Linear Range (nM)	LOD (nM)	Samples	Reference
MNA	GCE	DPV	$30–5.0\times10^{4}$	16	Hospital wastewater and tap water	1
PATP	Au electrode	LSV	3.6×10^{-6}–3.6	-	River water	2
PATP	GCE	SWV	0.01–100	1.86×10^{-3}	Tap and bottled water	3
NPEDMA	Au-coated glass	CV	1000−8000	686	Water	4
PATP	SPCE	EIS	0.1−10	0.002	PBS	5
Pyrrole	GCE	DPV	1×10^{-5}–1	3.8	Lake water	6
o-PD	Au-coated PET	SWV	0.5–500	0.18 and 0.41	PBS and Wastewater	**This work**

MNA: 6-mercaptonicotinic acid, PATB: p-aminothiophenol, NPEDMA: N-phenylethylene diamine methacrylamide, *o*-PD: o-Phenylenediamine, Au: Gold, GCE: Glassy carbon electrode, SPCE: Screen-printed carbon electrode, LSV: Linear sweep voltammetry, DPV: Differential pulse voltammetry.

References

(1) Yuan, L.; Zhang, J.; Zhou, P.; Chen, J.; Wang, R.; Wen, T.; Li, Y.; Zhou, X.; Jiang, H. Electrochemical sensor based on molecularly imprinted membranes at platinum nanoparticles-modified electrode for determination of 17β-estradiol. *Biosensors and Bioelectronics* **2011**, *29* (1), 29-33. DOI: 10.1016/j.bios.2011.07.058.

(2) Florea, A.; Cristea, C.; Vocanson, F.; Săndulescu, R.; Jaffrezic-Renault, N. Electrochemical sensor for the detection of estradiol based on electropolymerized molecularly imprinted polythioaniline film with signal amplification using gold nanoparticles. *Electrochemistry Communications* **2015**, *59*, 36-39. DOI: 10.1016/j.elecom.2015.06.021.

(3) Regasa, M. B.; Nyokong, T. Design and fabrication of electrochemical sensor based on molecularly imprinted polymer loaded onto silver nanoparticles for the detection of 17-β-estradiol. *Journal of Molecular Recognition* **2022**, *35* (10), e2978. DOI: 10.1002/jmr.2978.

(4) Des Azevedo, S.; Lakshmi, D.; Chianella, I.; Whitcombe, M. J.; Karim, K.; Ivanova-Mitseva, P. K.; Subrahmanyam, S.; Piletsky, S. A. Molecularly Imprinted Polymer-Hybrid Electrochemical Sensor for the Detection of β-Estradiol. *Industrial & Engineering Chemistry Research* **2013**, *52* (39), 13917-13923. DOI: 10.1021/ie302999j.

(5) Truong, T. N. L.; Van Toan, P.; Hao, N. Q. Using AuNPs-modified screen-printed electrode in the development of molecularly imprinted polymer for artificial bioreceptor fabrication to improve biosensor sensitivity for 17β-estradiol detection. *Advances in Natural Sciences: Nanoscience and Nanotechnology* **2019**, *10* (1), 015015. DOI: 10.1088/2043-6254/ab0d1b.

(6) Duan, D.; Si, X.; Ding, Y.; Li, L.; Ma, G.; Zhang, L.; Jian, B. A novel molecularly imprinted electrochemical sensor based on double sensitization by MOF/CNTs and Prussian blue for detection of 17β-estradiol. *Bioelectrochemistry* **2019**, *129*, 211-217. DOI: 10.1016/j.bioelechem.2019.04.014.
